# Development, characterization, and replication of proteomic aging clocks: Analysis of 2 population-based cohorts

**DOI:** 10.1371/journal.pmed.1004464

**Published:** 2024-09-24

**Authors:** Shuo Wang, Zexi Rao, Rui Cao, Anne H. Blaes, Josef Coresh, Rajat Deo, Ruth Dubin, Corinne E. Joshu, Benoit Lehallier, Pamela L. Lutsey, James S. Pankow, Wendy S. Post, Jerome I. Rotter, Sanaz Sedaghat, Weihong Tang, Bharat Thyagarajan, Keenan A. Walker, Peter Ganz, Elizabeth A. Platz, Weihua Guan, Anna Prizment

**Affiliations:** 1 Department of Laboratory Medicine and Pathology, University of Minnesota, Minneapolis, Minnesota, United States of America; 2 Division of Biostatistics and Health Data Science, School of Public Health, University of Minnesota, Minneapolis, Minnesota, United States of America; 3 Division of Hematology, Oncology and Transplantation, University of Minnesota, Minneapolis, Minnesota, United States of America; 4 Departments of Population Health and Medicine, New York University Glossman School of Medicine, New York, New York, United States of America; 5 Division of Cardiovascular Medicine, Perelman School of Medicine, University of Pennsylvania, Philadelphia, Pennsylvania, United States of America; 6 Division of Nephrology, University of Texas Southwestern Medical Center, Dallas, Texas, United States of America; 7 Department of Epidemiology, Johns Hopkins Bloomberg School of Public Health, Baltimore, Maryland, United States of America; 8 Sidney Kimmel Comprehensive Cancer Center at Johns Hopkins, Baltimore, Maryland, United States of America; 9 Alkahest Inc, San Carlos, California, United States of America; 10 Division of Epidemiology and Community Health, School of Public Health, University of Minnesota, Minneapolis, Minnesota, United States of America; 11 Division of Cardiology, Department of Medicine, Johns Hopkins University, Baltimore, Maryland, United States of America; 12 Institute for Translational Genomics and Population Sciences, The Lundquist Institute for Biomedical Innovation; Department of Pediatrics, Harbor-UCLA Medical Center, Torrance, California, United States of America; 13 Laboratory of Behavioral Neuroscience, National Institute on Aging, National Institutes of Health, Baltimore, Maryland, United States of America; 14 Department of Medicine, University of California, San Francisco, California, United States of America; National Cancer Institute, UNITED STATES OF AMERICA

## Abstract

**Background:**

Biological age may be estimated by proteomic aging clocks (PACs). Previous published PACs were constructed either in smaller studies or mainly in white individuals, and they used proteomic measures from only one-time point. In this study, we created de novo PACs and compared their performance to published PACs at 2 different time points in the Atherosclerosis Risk in Communities (ARIC) study of white and black participants (around 75% white and 25% black).

**Medthods and findings:**

A total of 4,712 plasma proteins were measured using SomaScan in blood samples collected in 1990 to 1992 from 11,761 midlife participants (aged 46 to 70 years) and in 2011 to 2013 from 5,183 late-life participants (aged 66 to 90 years). The de novo ARIC PACs were constructed by training them against chronological age using elastic net regression in two-thirds of healthy participants in midlife and late life and validated in the remaining one-third of healthy participants at the corresponding time point. We also computed 3 published PACs. We estimated age acceleration for each PAC as residuals after regressing each PAC on chronological age. We also calculated the change in age acceleration from midlife to late life. We examined the associations of age acceleration and change in age acceleration with mortality through 2019 from all-cause, cardiovascular disease (CVD), cancer, and lower respiratory disease (LRD) using Cox proportional hazards regression in participants (irrespective of health) after excluding the training set. The model was adjusted for chronological age, smoking, body mass index (BMI), and other confounders. We externally validated the midlife PAC using the Multi-Ethnic Study of Atherosclerosis (MESA) Exam 1 data. The ARIC PACs had a slightly stronger correlation with chronological age than published PACs in healthy participants at each time point. Associations with mortality were similar for the ARIC PACs and published PACs. For late-life and midlife age acceleration for the ARIC PACs, respectively, hazard ratios (HRs) per 1 standard deviation were 1.65 and 1.38 (both *p* < 0.001) for all-cause mortality, 1.37 and 1.20 (both *p* < 0.001) for CVD mortality, 1.21 (*p* = 0.028) and 1.04 (*p* = 0.280) for cancer mortality, and 1.68 and 1.36 (both *p* < 0.001) for LRD mortality. For the change in age acceleration, HRs for all-cause, CVD, and LRD mortality were comparable to the HRs for late-life age acceleration. The association between the change in age acceleration and cancer mortality was not significant. The external validation of the midlife PAC in MESA showed significant associations with mortality, as observed for midlife participants in ARIC. The main limitation is that our PACs were constructed in midlife and late-life participants. It is unknown whether these PACs could be applied to young individuals.

**Conclusions:**

In this longitudinal study, we found that the ARIC PACs and published PACs were similarly associated with an increased risk of mortality. These findings suggested that PACs show promise as biomarkers of biological age. PACs may be serve as tools to predict mortality and evaluate the effect of anti-aging lifestyle and therapeutic interventions.

## Abstract 2

Shuo Wang and colleagues, apply machine learning to develop proteomic aging clocks from 5000 proteins sampled from participants at mid- and late-life and explore the impact of lifestyle factors and medical conditions on biological aging and the association with mortality.

## Author summary

**Why was this study done?:**

- Proteomic aging clocks (PACs), which are protein-based aging measures, may be used to estimate an individual’s biological age and have been shown to be associated with mortality and other health conditions. However, previous published PACs were developed in relatively small studies that mainly included white individuals.
- The aim of this study was to develop new PACs in a large population-based prospective cohort of white and black participants that have approximately 5,000 proteins measured in blood samples collected at midlife and late life and to compare the performance of new and published PACs in midlife and late-life participants.

**What did the researchers do and find?:**

- We applied machine learning to develop new PACs among a group of healthy participants at midlife (aged 46 to 70 years) and late life (aged 66 to 90 years) in a prospective population-based cohort study and validated these clocks in the remaining participants.
- We examined the association between the new PACs and mortality among 8,768 midlife participants and 4,553 late-life participants irrespective of their health status. We also examined the association between the change in PACs from midlife to late life and mortality. We found that the midlife PAC and the late-life PAC and the change in PACs from midlife to late life were associated with increased mortality by 20 to 68 percent.
- We examined whether midlife lifestyle factors and medical conditions were associated with higher biological age in late life. We found that smoking, and having high BMI, diabetes, hypertension, cardiovascular disease (CVD), and lower eGFR in midlife were associated with a higher biological age in late life.

**What do these findings mean?:**

- These findings suggest that PACs show promise as a biomarker of biological age. PACs could be applied in clinical trials that evaluate the effect of anti-aging lifestyle and therapeutic interventions.
- The notable contribution of modifiable risk factors, such as smoking and BMI, and cardiometabolic disorders to biological aging as identified by this study, may help to further inform public policies targeted at prevention.
- The limitation of this study is that our PACs were created in midlife and late-life participants. It is unknown whether these PACs could be applied to young individuals.

## Introduction

An individual’s extent of aging, i.e., how far an individual is into the aging process, cannot be sufficiently measured by chronological age as individuals develop physiological dysregulations at different chronological ages [1,2]. To better understand the extent of aging, researchers introduced a term called “biological age” to capture how far individuals are into their aging process. Biological age, according to the definition proposed by Baker and Sprott, is characterized by the “biological parameter[s] of an organism, either alone or in some multivariate composite that will, in the absence of disease, better predict functional capability at some late age than will chronological age” [3].

To estimate a person’s biological age, researchers have developed metrics called aging clocks using epigenetic, transcriptomic, metabolomic, proteomic, and other biomarkers [4]. Aging clocks are strongly correlated with chronological age in healthy individuals. However, in individuals with comorbidities or predisposing conditions, aging clocks deviate from chronological age because these conditions impact levels of age-associated biomarkers [5,6]. Studies show that aging clocks may be used to identify individuals who have a positive deviation of biological age from their chronological age (called age acceleration) that may predict their future risk of age-related conditions [5–7]. In addition, aging clocks may also track the effectiveness of anti-aging interventions in clinical trials [5,8–10].

The most studied aging clocks are epigenetic clocks, such as Horvath clock, Hannum clock, DNAm PhenoAge, and GrimAge [11–14]. However, there is a lack of understanding of the underlying mechanisms of aging-related changes in DNA methylation sites. It remains unclear what aspects of aging those clocks reflect [15]. Recently, new assays, such as the SomaScan assay—a modified aptamer-based technology [16–18]—that measure thousands of proteins in a small blood sample simultaneously have been developed. These assays make it possible to construct proteomic aging clocks (PACs) [5–7,19]. The strength of PACs is that they include proteomic-based biomarkers, an intermediate phenotype that is most proximal to age-related diseases, and thus may provide more accurate information on aging and age-related pathologies [5,20]. Importantly, proteins serve as a target in 96% of FDA-approved drugs [21]. Therefore, in addition to predicting biological age and risk of diseases, proteins comprising PACs, if causal, hold promise as targets of anti-aging drugs. Targeting age-related processes or pathological manifestations instead of a single disease is advantageous as this approach may simultaneously reduce the development of multiple age-related diseases and potentially prolong health span.

Several PACs have been developed using SomaScan assays, such as the PACs created by Lehallier [2020] (*N* = 3,301, aged 18 to 76 years) [6], Tanaka [2018] (*N* = 240, aged 22 to 93 years) [5], and Sathyan [2020] (*N* = 1,025, aged 65 to 95 years) [19]. The descriptions of those published PACs, including the number of proteins used to construct those PACs, are presented in **S1 Table**. Although those published PACs showed high correlations with chronological age, they were developed either in relatively small studies or in studies included individuals of European descent [5,6,19,22]. However, proteins associated with age and age-related diseases vary by race and socioeconomic status [23–25]. Moreover, previously published PACs were constructed using a one-time measure. Thus, it is necessary to develop PACs in a large longitudinal study of diverse individuals and examine if the change in PACs over time is associated with mortality independent of chronological age, smoking, and other lifestyle and behavioral factors.

In this study, we developed new PACs in participants followed from midlife and late life and examined their associations with mortality within a large population-based prospective cohort of white and black, men and women, in the Atherosclerosis Risk in Communities (ARIC) study. In ARIC, about 5,000 plasma proteins were measured using the SomaScan assay (v4.0) from plasma samples collected at 2 different times (20 years apart). We aimed to compare the midlife and late-life ARIC PACs developed in healthy participants (without major age-associated diseases) with the published Lehallier’s, Tanaka’s, and Sathyan’s PACs. We compared their correlations with chronological age and their associations with mortality from all-cause, cardiovascular disease (CVD), cancer, and lower respiratory disease (LRD). In addition, using protein data measured at 2 different time points, we examined whether the change in PACs from midlife to late life was associated with premature mortality. We also validated the midlife ARIC PAC in the Multi-Ethnic Study of Atherosclerosis (MESA) study by examing its correlation with chronological age and its association with mortality.

## Methods

### Study population

This study included white and black, men and women, participants of the ongoing ARIC study (RRID: SCR_021769), which was initiated in 1987 [26,27]. At Visit 1 (1987 to 1989), 15,792 volunteers aged 45 to 64 years were recruited from 4 US study centers, Washington County, Maryland; suburban Minneapolis, Minnesota; Jackson, Mississippi; and Forsyth County, North Carolina. Participants in the Minnesota and Maryland centers were primarily white and the recruitment in Mississippi was restricted to black residents. ARIC was approved by institutional review boards at each participating center and all study participants provided written informed consent. To date, 10 visits have been completed [26]. ARIC participants have received follow-up telephone calls annually from 1987 to 2012 and semi-annually after 2012, with response rates of 83% to 99% for the follow-up calls among living participants who have not withdrawn consent to be contacted [27]. There is also continuous surveillance of local hospitals and linkage to the National Death Index (NDI).

### Plasma collection

In this study, we used plasma samples collected at Visit 2 (1990 to 1992) from 11,761 participants aged 46 to 70 years (midlife) and at Visit 5 (2011 to 2013) from 5,183 participants aged 66 to 90 years (late life). The blood sample collection, processing, and storage in ARIC was designed to minimize the spontaneous biochemical reactions after blood collection and is consistent with the recommended practice for proteomics data analysis in epidemiological studies [16,28,29]. After venipuncture, blood samples were put immediately in an ice water bath. Centrifugation was performed within 10 min after venipuncture at room temperature (15 to 25°C). After centrifugation, the aliquots were stored at −80°C within 90 min from venipuncture and were unthawed before this analysis.

### Protein measurement and quality control

Plasma samples were analyzed using a SOMAmer (Slow Off-rate Modified Aptamers) based capture array called SomaScan by Somalogic (Boulder, Colorado, United States of America) [18,30–32]. The SomaScan platform uses single-stranded modified DNA-based aptamers to capture conformational protein epitopes. The description of the SomaScan assay and the data normalization process have been described previously [16,17,32].

Among the 5,284 available aptamers, we excluded aptamers with a Bland–Altman coefficient of variation (CVBA) greater than 50% or a variance of less than 0.01 on the log scale, or binding to mouse Fc-fusion, contaminants, or non-proteins [33]. After the exclusion, 4,955 aptamers were included (at Visit 2 and Visit 5) which corresponded to 4,712 proteins. About 5% of proteins had more than 1 aptamer binding to the same protein. Each aptamer was treated as a variable in the construction of PACs. The CVBA for split samples was 6% at Visit 2 and 7% at Visit 5. Protein measures, reported as relative fluorescent units (RFUs), were log2-transformed to correct for skewness.

### Identifying healthy participants

**Fig 1:**
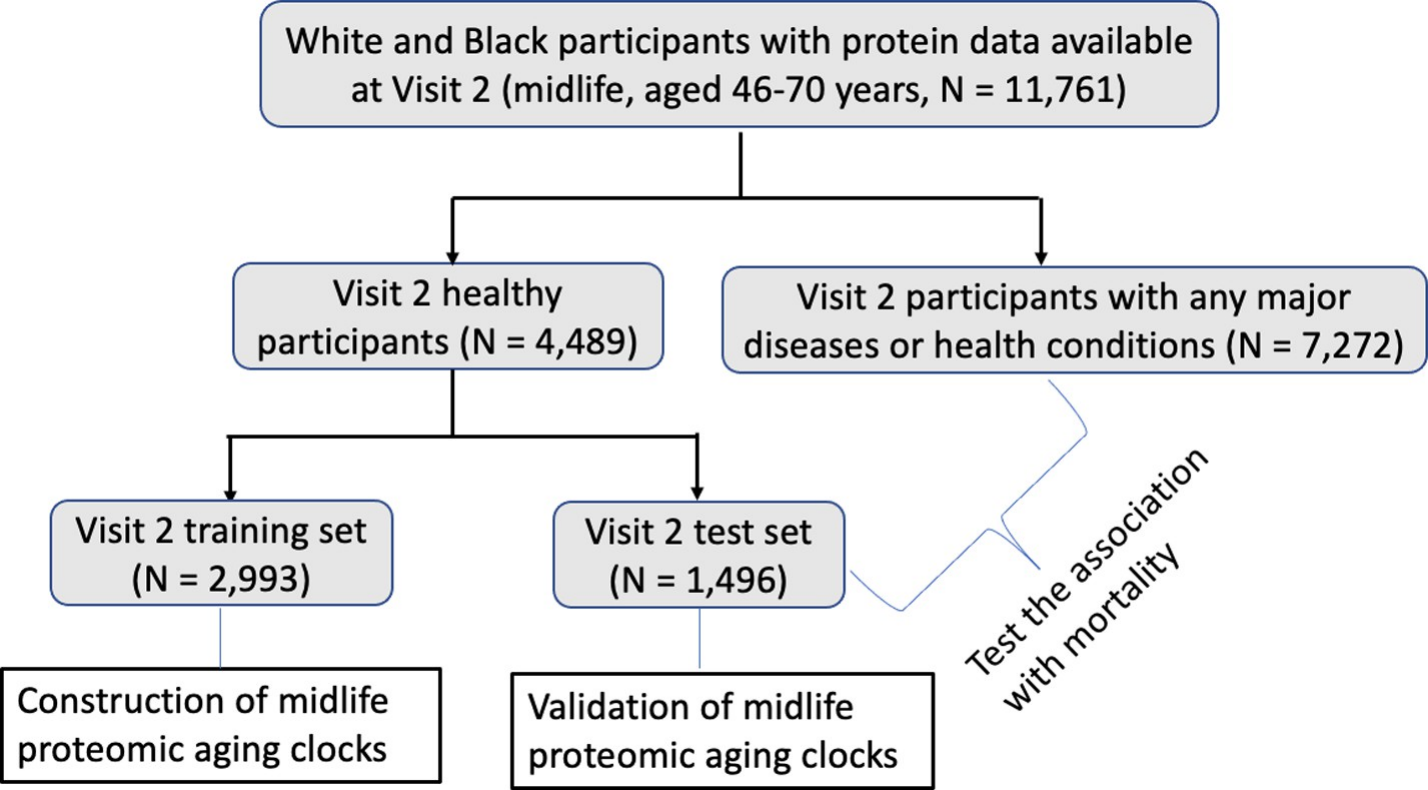
Study population at Visit 2 (1990–1992; midlife, aged 46–70 years); ARIC. The midlife ARIC PAC was constructed in a group of health participants in the training set and its asssociation with mortality was examined in all remaining pariticpants irrespective of health. ARIC, Atherosclerosis Risk in Communities; PAC, proteomic aging clock.

**Fig 2:**
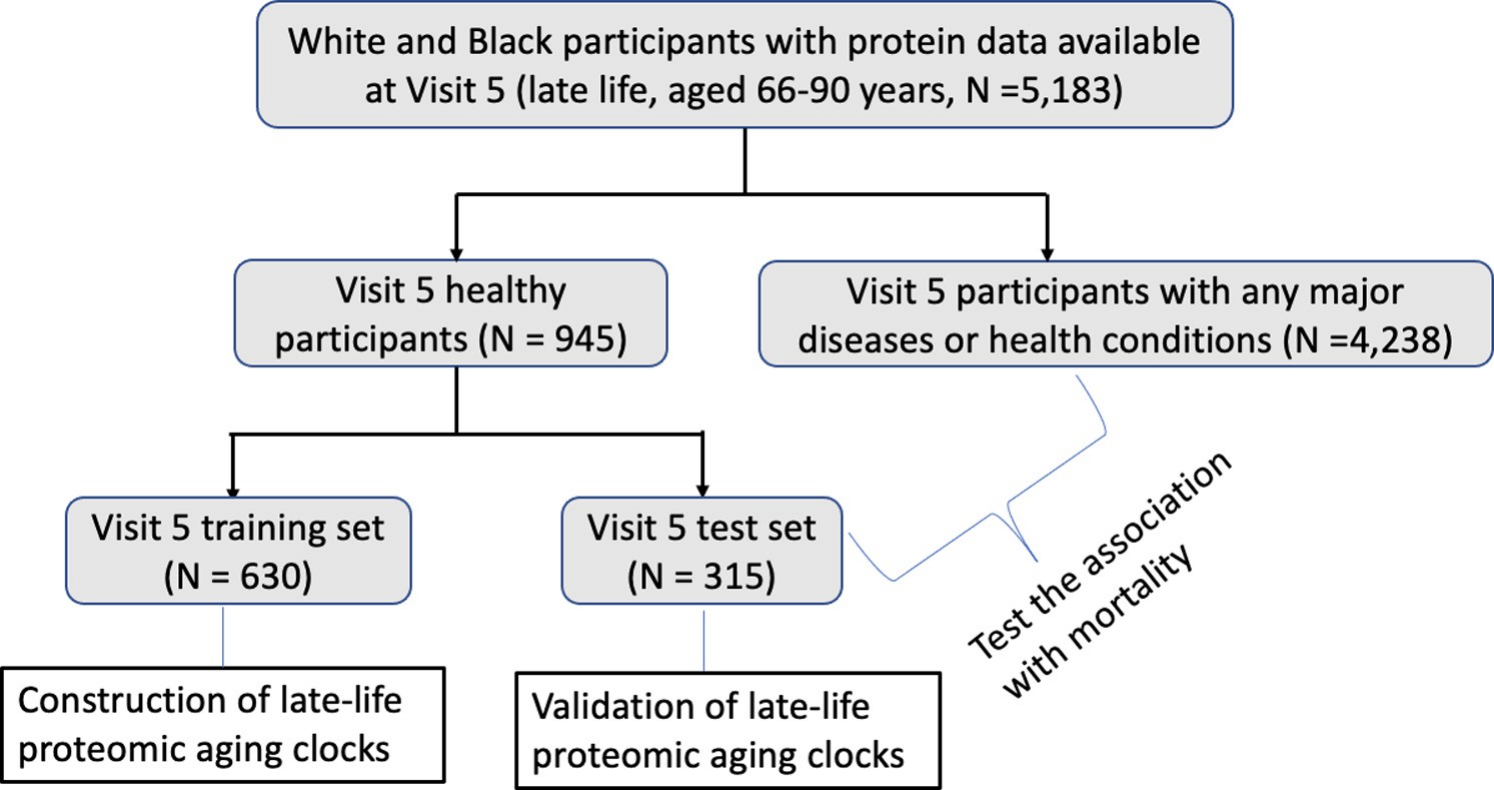
Study population at Visit 5 (2011–2013; late life, aged 66–90 years); ARIC. The late-life ARIC PAC was constructed in a group of health participants in the training set and its asssociation with mortality was examined in all remaining pariticpants irrespective of health. ARIC, Atherosclerosis Risk in Communities; PAC, proteomic aging clock.

In this study, we created the midlife (Visit 2) and late-life (Visit 5) ARIC PACs in “healthy participants” defined as participants without major age-associated diseases that are linked to premature mortality. Specifically, abnormal kidney function (i.e., estimated glomerular filtration rate (eGFR) less than 60 mL/min/1.73 m^2^), cancer, chronic obstructive pulmonary disease (COPD), CVD (heart failure, definite or probable stroke, or coronary heart disease [34,35]), diabetes, and hypertension. The definitions and assessments of these major diseases in ARIC and the detailed process of identifying healthy participants are described in S1 Appendix. We identified 4,489 midlife healthy participants at Visit 2 (38.2% of all Visit 2 participants, Fig 1) and 945 late-life healthy participants at Visit 5 (18.2% of all Visit 5 participants, Fig 2).

### Assessment of mortality and other characteristics of interest

Deaths were ascertained through annual (semi-annual since 2012) follow-up telephone calls to participants or their proxies, surveillance of local hospitals, state records, and linkage to NDI through December 31, 2017 for participants in Mississippi or through December 31, 2019 for participants in other centers [36]. All-cause mortality was defined as death resulting from any cause. CVD mortality, cancer mortality, and LRD mortality were defined based on the underlying cause of death using *International Classification of Diseases*, *Ninth Revision*, codes (ICD-9 codes) 390–459 or *International Classification of Diseases*, *Tenth Revision*, codes (ICD-10 codes) I00–I99 for CVD deaths, ICD-9 codes 140–239 or ICD-10 codes C00-C97 for cancer deaths, and ICD-9 codes 466 and 480–519 or ICD-10 codes J10-J98 for LRD deaths.

Other characteristics of interest included demographic and lifestyle characteristics, and medical conditions. Namely chronological age, sex, race, study center, education, smoking status, pack-years of smoking, alcohol intake, body mass index (BMI), physical activity, aspirin use, hormone replacement therapy (HRT) in females (only at Visit 2; this variable is not available at Visit 5), diabetes, hypertension, CVD, and eGFR [26]. Education attainment was collected at Visit 1. Physical activity was collected at Visit 1 (1987 to 1989) (used as physical activity at Visit 2 (1990 to 1992) in this study) and Visit 5. The other variables listed above were collected at both Visit 2 and Visit 5. Detailed procedures for assessing these characteristics are described in S1 Appendix.

### The MESA study

The MESA study is a prospective cohort of white, black, Hispanic, and Chinese participants. At Exam 1 (2000 to 2002), 6,814 participants aged 45 to 84 years were recruited from 6 field centers: Baltimore, Maryland; Chicago, Illinois; Forsyth County, North Carolina; Los Angeles, California; New York, New York; and Minneapolis, Minnesota. Each field center recruited approximately equal numbers of females and males from 2 or more of the racial/ethnic groups [37]. Details of the MESA study are available at the MESA website (https://www.mesa-nhlbi.org).

MESA has measured around 7,000 proteins using the SomaScan assay (v4.1), which includes all the proteins from the SomaScan assay (v4.0) used in ARIC, from the blood samples collected at Exam 1. Participants have been followed for up to 18 years with systematic ascertainment and adjudication of mortality. Deaths from CVD, cancer, and LRD were classified according to the underlying cause (ICD-10). Institutional review boards approved the study at each field center, and all participants provided written informed consent [37].

### Statistical analysis

#### Development of PACs

To construct ARIC PACs in midlife (Visit 2) and late life (Visit 5), we randomly selected two-thirds of healthy participants at each visit and used them as the training set at the corresponding visit. The remaining one-third of healthy participants were used as the test set (Figs 1 and 2). We utilized the training set to train PACs against chronological age and obtain the appropriate hyperparameter values and weight for each aptamer: $chronological\;age=\beta_{0}+\sum_{i=1}^{n}\beta_{i}\times aptamer_{i}$, where *aptamer*_*i*_ is the level of the *ith* aptamer. We used the test set to examine the Pearson correlation (r) between PAC and chronological age and median absolute error (MAE) to validate each PAC.

#### Construction of midlife PACs in the Visit 2 training set

Using the Visit 2 training set, following the methodology used in the previous studies [5,11,19], we constructed the midlife ARIC PAC using elastic net regression (alpha = 0.5) and with log2-transformed proteins. We also developed several PACs using other alpha values, and all these PACs were highly correlated with each other and with the midlife ARIC PAC (r > 0.94). We finally chose an alpha value of 0.5 because this value was used in all the previous studies. Following the methodology used in previous studies, lambda value was selected based on 10-fold cross-validation in the training set. We chose elastic net regression because it combines the penalties from both Lasso and Ridge regressions, and most previous aging clocks, including PACs and epigenetic clocks, were constructed using elastic net regression. Using the Visit 2 proteomics data, we also trained 4 other midlife PACs by applying different penalized regression methods and various protein transformations (described in S2 Table). For instance, one of the created PACs accounted for the potential nonlinear associations between proteins and chronological age by including both the square and cubic terms of each aptamer. Those 4 PACs were strongly correlated (r ≥ 0.97) with the midlife ARIC PAC that was constructed using the simplest protein transformation (S3 Table). Therefore, the simplest ARIC PAC was used for further investigation.

In addition to the midlife ARIC PAC, we also computed 3 published PACs in midlife: Lehallier’s [6], Tanaka’s [5], and Sathyan’s PACs [19]. In our study, we computed Sathyan’s PAC (a published PAC developed using the same panel of SomaScan assay as in ARIC) using the published weights. For Lehallier’s and Tanaka’s PACs, we had to estimate ARIC weights specific to these PACs because ARIC did not include all the aptamers reported in these PACs and the use of published weights is not suitable in such case (S1 Table). To estimate ARIC weights, in the training set, we applied Ridge regression to train the available aptamers in ARIC against chronological age. The lambda value for Ridge regression was selected based on 10-fold cross-validation in the traning set. We conducted a sensitivity analysis to test whether using Ridge regression to compute published PACs influences the performance of those PACs by applying 2 approaches to compute Sathyan’s PAC. In the first approach, we computed Sathyan’s PAC using the published weights. In the second approach, we estimated ARIC weights for Sathyan’s PAC by applying Ridge regression. We found that Sathyan’s PAC computed using these 2 approaches showed the same associations with mortality. Therefore, we conclude that using Ridge regression to compute published PACs does not influence the performance of PACs.

#### Construction of late-life PACs in the Visit 5 training set

Because hypertension is one of the most common conditions in older persons in the United States [38], to construct the late-life ARIC PAC, we additionally included participants with controlled hypertension as healthy participants. Controlled hypertension was defined as the measured diastolic blood pressure being below 90 and the measured systolic blood pressure being below 140 while the participant is on medication [39]. Adding these participants increased the number of healthy participants by 95% (462 participants) but did not change the PAC’s performance as shown in S4 Table.

Using the Visit 5 training set, we constructed the late-life ARIC PAC using elastic net regression, the same approach as for the midlife ARIC PAC. In addition to the late-life ARIC PAC, we computed the late-life Lehallier’s and Tanaka’s PACs using ARIC weights estimated using the Visit 5 training set by applying Ridge regression as discussed above and we computed the Sathyan’s PAC using the published weights.

#### Internal validation of PACs and examining associations with mortality

In the remaining 8,768 participants (including the healthy participants in the test set and participants with any major diseases or health conditions) at Visit 2 (Fig 1) and 4,553 participants at Visit 5 (Fig 2) after excluding the training set, we computed PACs at the corresponding visit as the weighted sum of proteins determined in the training set. We internally validated each PAC in the test set of healthy participants at the corresponding visits by computing the Pearson correlation between PAC and chronological age at that visit and MAE.

In all the remaining participants at each visit, to capture the PACs’ effects independent of chronological age, we created age acceleration for each PAC as residuals after regressing PAC on chronological age [40]. Demographic and lifestyle characteristics, and medical conditions were examined across quartiles of age acceleration as mean (SD) or percentage (%). To further investigate PACs, we examined the associations between PACs and mortality. We used Cox proportional hazards regression to calculate hazard ratios (HRs) and 95% confidence intervals (CIs) for mortality from all-cause, CVD, cancer, and LRD with age acceleration. For the associations with CVD mortality, cancer mortality, and LRD mortality, deaths from other causes were treated as competing events using the Fine and Gray method [41,42]. We modeled age acceleration as a continuous variable because there was no evidence of nonlinearity observed when we applied cubic splines. For each participant, the total person-years were determined from the date of blood collection (at Visit 2 or Visit 5, depending on the analysis) until death, censoring, or the end of follow-up (either December 31, 2017 for participants from Mississippi or December 31, 2019 for participants from other centers), whichever occurred first. The proportional hazards assumption, examined by the graphical methods using log-log survival curves with age acceleration dichotomized at the median, was not violated in any regression models. The model was adjusted for chronological age, sex, joint terms for race and study center (black participants from Mississippi; black participants from any other centers; white participants from Maryland; white participants from North Carolina; and white participants from Minnesota), education, BMI, smoking status, pack-years of smoking, alcohol intake, physical activity, HRT (at Visit 2 only), diabetes, hypertension, CVD, and eGFR (fully adjusted model). These variables were associated with either age acceleration or risk of mortality. To confirm these variables as potential confounders, we computed the magnitude of R squared by regressing age acceleration for both the midlife and late-life ARIC PACs on these variables at the corresponding visits in the model adjusted for chronological age (S5 Table). We did not adjust for aspirin use because aspirin use had no association with midlife or late-life age acceleration for the ARIC PACs and aspirin use explained <0.0015 of variance in both midlife and late-life age acceleration (S5 Table). In this study, we found that HRs (95% CIs) for mortality were the same in the age-adjusted and fully adjusted models. Thus, we reported results for the fully adjusted model.

We also examined whether the change in age acceleration from midlife (Visit 2) to late life (Visit 5), computed as the age acceleration for the late-life ARIC PAC minus the age acceleration for the midlife ARIC PAC, was associated with mortality using Cox proportional hazard regression. For each participant, the total person-years was determined from Visit 5 date until death, censoring, or the end of follow-up. For this analysis, we additionally adjusted for midlife age acceleration. Also, we examined whether the associations with mortality were modified by midlife age acceleration (continuous variable) using a multiplicative term between the change in age acceleration and midlife age acceleration. For the change in age acceleration, we only examined the change based on the ARIC PACs because the ARIC PACs and published PACs showed similar associations with all mortality types at each visit.

In addition to studying the associations with mortality, we examined if midlife lifestyle characteristics and medical conditions (Visit 2) were associated with late-life age acceleration (Visit 5). This analysis was conducted using multivariable linear regression and midlife participants’ lifestyle characteristics and medical conditions including: chronological age, sex, race, education, BMI, smoking status, pack-years of smoking, alcohol intake, physical activity (at Visit 1), HRT use, diabetes, hypertension, CVD, and eGFR were included into the model simultaneously.

We did a sensitive analysis to test whether the exclusion of the training set influenced the associations between PACs and mortality because the distribution of outcome was slightly changed after excluding the training set. We examined this by comparing the associations between age acceleration for Sathyan’s PAC and mortaliy in all participants and in participants after excluding the training set. We used Sathyan’s PAC rather than other published PACs because all the proteins reported in Sathyan’s PAC were measured in ARIC and we were able to calculate Sathyan’s PAC using published weights.

We also examined whether sex, race, or chronological age (in tertiles) modified the associations of age acceleration with all-cause mortality, CVD mortality, and cancer mortality by including a multiplicative term between age acceleration and the variable of interest in the corresponding models. We did not examine LRD mortality due to the limited number of LRD deaths. Additionally, we examined the association between age acceleration for the midlife ARIC PAC and the 10-year risk of death as this may be important for clinical screening. We tested the 10-year risk for midlife PAC only, because the follow-up period starting from late life was less than 10 years. Here, we examined the midlife ARIC PAC only, because the ARIC PACs and published PACs showed similar associations with all mortality types. Moreover, we applied the midlife ARIC PAC to late-life participants and applied the late-life ARIC PAC to midlife participants and then examined the asosociation with all-cause mortality.

#### External validation of the midlife ARIC PAC in the MESA study

We applied the midlife ARIC PAC to 4,288 participants who had proteomic data and aged 46 to 70 years at Exam 1 and examined the correlation between the midlife ARIC PAC and chronological age. We then calculated age acceleration for the midlife ARIC PAC and examined the associations of age acceleration with mortality from all-cause, CVD, cancer, and LRD until 2018. Moreover, we examined the association with all-cause mortality stratified by race/ethnicity (white, black, Chinese, and Hispanic). We did not examine the association with CVD, cancer, and LRD mortality stratified by race/ethnicity due to the limited numbers of deaths in Chinese and Hispanic participants. We adjusted for the same covariates in the model as in ARIC. We did not apply the late-life ARIC PAC to MESA Exam 1 because the late-life ARIC PAC was developed in a population on average older than the MESA Exam 1 participants. We also applied Lehallier’s PAC to MESA participants and examined its association with mortality. We applied only 1 published PAC in MESA because all the published PACs showed similar associations with mortality in ARIC. We selected Lehallier’s PAC rather than other published PACs because Lehallier’s PAC showed the highest correlation with chronological age in the published paper (S1 Table).

In this study, PACs were constructed using R (version 4.1.2, package “glmnet”), and all the other analyses were performed using SAS 9.4 (RRID: SCR_008567). Statistical significance was considered if a two-sided *p*-value <0.05. The formulas (intercept and weights for proteins) for the PACs used in this study can be found in S2 Appendix.

## Results

### Midlife PACs

**Fig 3:**
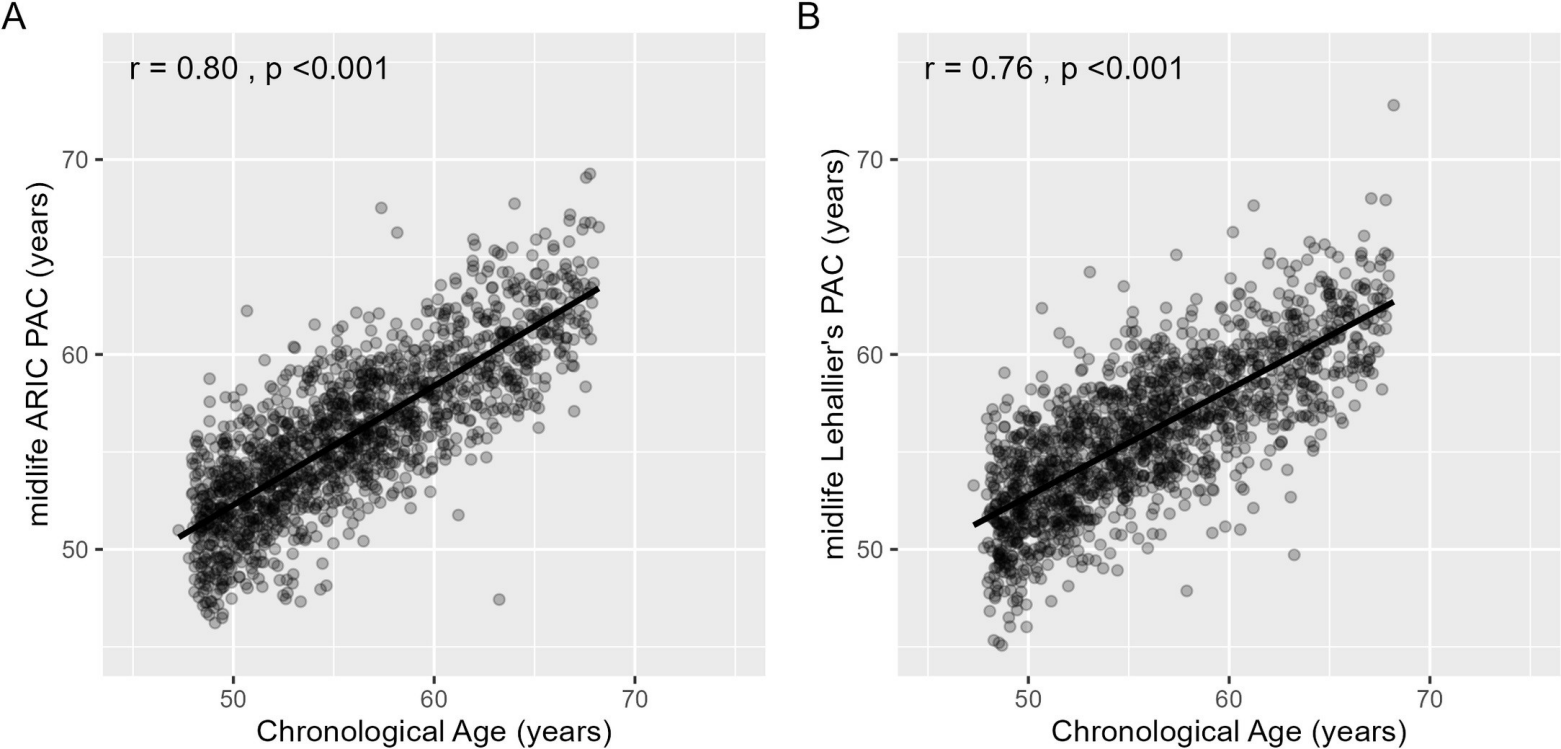
Pearson correlation (r) between the midlife ARIC PAC and Lehallier’s PAC and chronological age in the Visit 2 test set of healthy participants. The x-axis depicts chronological age. The y-axis represents PAC. (A) The midlife ARIC PAC was constructed using healthy participants from ARIC. The correlation between the midlife ARIC PAC and chronological age was 0.80 (*p* < 0.001). (B) Lehallier’s PAC was computed using ARIC weights obtained from Ridge regression based on proteins available in ARIC. The correlation between midlife Lehallier’s PAC and chronological age was 0.76 (*p* < 0.001). ARIC, Atherosclerosis Risk in Communities; PAC, proteomic aging clock.

Elastic net regression selected 788 aptamers for the midlife ARIC PAC (Table 1), and 67% (*N* = 524) of these 788 patamers were significantly associated (*p* < 0.050) with chronological age in a single protein model. In our study, we trained our PACs using elastic net regression that included all 5,000 proteins in one model. It is possible that the significance of each individual protein may change after accounting for other proteins included into the same model. Unfortunately, there is no way to check *p*-values in elastic net regression as the model provides only weights for proteins but not *p*-values, making it difficult to interpret statistical significance [43]. In the Visit 2 test set, the midlife ARIC PAC was correlated with chronological age (r = 0.80, MAE = 2.19 years, Table 1 and Fig 3A). Of the 3 midlife published PACs, Lehallier’s PAC (r = 0.76, *p* < 0.001, Table 1 and Fig 3B) had a slightly higher correlation with chronological age than Tanaka’s (r = 0.66, *p* < 0.001) and Sathyan’s PACs (r = 0.58, *p* < 0.001) (S6 Table and S1 Fig). The midlife ARIC PAC was strongly correlated with the midlife Lehallier’s (r = 0.89, *p* < 0.001), Tanaka’s (r = 0.77, *p* < 0.001), and Sathyan’s PACs (r = 0.71, *p* < 0.001) (S3 Table).

**Table 1:** Pearson correlations between the midlife and late-life ARIC PACs and Lehallier’s PACs and chronological age and MAE, ARIC.

***The midlife ARIC PAC and Lehallier’s PAC; Visit 2 (N = 2*,*993 in training set*, *N = 1*,*496 in test set)***		
	midlife ARIC PAC	midlife Lehallier’s PAC
Number of aptamers in PAC	788	415
Hyperparameter value (lambda)	0.11	1.03
Correlation in the training set^a^	0.92 (*p* < 0.001)	0.82 (*p* < 0.001)
Correlation in the test set^a^	0.80 (*p* < 0.001)	0.76 (*p* < 0.001)
MAE in the training set^a^	1.50	2.13
MAE in the test set^a^	2.19	2.39
***The late-life ARIC PAC and Lehallier’s PAC; Visit 5 (N = 630 in training set*, *N = 315 in test set)***		
	late-life ARIC PAC	late-life Lehallier’s PAC
Number of aptamers in PAC	135	415
Hyperparameter value (lambda)	0.46	4.42
Correlation in the training set^a^	0.84 (*p* < 0.001)	0.84 (*p* < 0.001)
Correlation in the test set^a^	0.71 (*p* < 0.001)	0.63 (*p* < 0.001)
MAE in the training set^a^	1.47	1.71
MAE in the test set^a^	2.36	2.37

^a^Among healthy participants at Visit 2 and Visit 5, we randomly selected two-thirds of healthy participants at each visit and used them as the training set at the corresponding visit; the remaining one-third of healthy participants at each visit was used as the test set at the corresponding visit.

ARIC, Atherosclerosis Risk in Communities; MAE, median absolute error; PAC, proteomic aging clock.

After excluding the Visit 2 training set, the remaining 8,768 participants in midlife were on average 58.1 ± 5.7 years old, 54.6% were female, and 27.1% self-identified as black. Distributions of midlife characteristics (Visit 2) across quartiles of midlife age acceleration are shown in Tables 2 and S7. Among the remaining participants in midlife, the range of age acceleration was from −14.0 to +24.2 years for the midlife ARIC PAC. The distributions of characteristics including HRT use, CVD, and eGFR were in the same direction across age acceleration for the midlife ARIC and published PACs (Tables 2 and S7). However, the distributions of sex, race, education, BMI, current smoking, aspirin use, hypertension, and diabetes were different across different PACs (Tables 2 and S7). The difference may be because different PACs capture different aspects of aging.

**Table 2:** Midlife participants’ characteristics across quartiles of age acceleration for the midlife ARIC PAC and Lehallier’s PAC; ARIC.

	Midlife ARIC PAC					Midlife Lehallier’s PAC				
	Q1 (*N* = 2,192)	Q2 (*N* = 2,192)	Q3 (*N* = 2,192)	Q4 (*N* = 2,192)	*P*-value^c^	Q1 (*N* = 2,192)	Q2 (*N* = 2,192)	Q3 (*N* = 2,192)	Q4 (*N* = 2,192)	*P*-value^c^
Age acceleration (min to max), years	−14.0 to −1.9	−1.8 to −0.2	−0.3 to +1.7	+1.8 to +24.2		−15.1 to −2.0	−1.9 to −0.2	−0.1 to +1.8	+1.9 to +26.5	
Mean age acceleration, years	−3.44	−1.04	0.68	3.79		−3.58	−1.04	0.77	3.84	
Chronological age, years (SD)	58.3 (5.8)	57.9 (5.8)	57.8 (5.7)	58.3 (5.6)	0.071	58.2 (5.8)	58.1 (5.8)	58.1 (5.6)	58.1 (5.6)	0.884
Female, %	55.6	51.6	55.4	55.8	0.015	53.2	53.2	55.5	56.5	0.060
White, %	72.8	75.7	75.2	67.9	<0.001	69.8	75.6	74.2	72.2	<0.001
Education, %										
Less than high school	21.8	21.8	22.9	30.0	<0.001	22.0	22.9	24.9	26.6	<0.001
High school/vocational	41.1	40.9	43.2	40.6		39.8	42.4	40.9	42.6	
College	37.1	37.4	33.9	29.3		38.2	34.6	34.1	30.7	
BMI, kg/m^2^ (SD)	28.2 (5.1)	28.3 (5.1)	28.3 (5.6)	28.6 (6.3)	0.127	28.5 (5.3)	28.4 (5.4)	28.2 (5.5)	28.4 (5.9)	0.579
Smoking status, %										
Current smoker	22.3	23.2	22.4	24.2	0.062	21.6	23.3	23.6	23.4	0.055
Former smoker	36.6	38.4	39.1	39.6		36.8	38.3	38.5	39.9	
Never smoker	41.1	38.4	38.6	36.2		41.5	38.4	37.8	36.5	
Pack-years of smoking among ever smokers, pack-years (SD)	28.5 (22.8)	30.4 (23.4)	29.9 (22.0)	32.5 (24.1)	<0.001	28.4 (22.3)	29.5 (23.1)	30.3 (23.0)	32.9 (23.7)	<0.001
Alcohol intake, %										
Current drinker	57.4	58.2	54.9	48.3	<0.001	54.8	56.8	55.9	51.2	0.002
Former drinker	21.6	20.9	20.8	26.5		22.7	20.4	21.2	25.3	
Never drinker	20.9	20.9	24.3	25.2		22.5	22.6	22.7	23.5	
Physical activity^a^, scores (SD)	2.48 (0.8)	2.45 (0.8)	2.42 (0.8)	2.33 (0.8)	<0.001	2.46 (0.8)	2.44 (0.8)	2.41 (0.8)	2.38 (0.8)	0.025
Aspirin use in the preceding 2 weeks, %	51.1	51.2	54.5	52.7	0.075	48.7	53.1	52.4	55.3	<0.001
Ever user of HRT (females only), %	50.2	47.0	41.2	36.3	<0.001	48.8	45.9	42.7	37.3	<0.001
Diabetes^b^, %	15.1	16.9	20.1	29.2	<0.001	17.0	16.4	20.2	27.8	<0.001
Hypertension^b^, %	42.6	46.2	49.4	55.8	<0.001	44.9	48.4	47.4	53.4	<0.001
CVD^b^, %	11.8	13.9	16.4	21.5	<0.001	11.8	13.7	15.6	22.8	<0.001
eGFR, mL/min/1.73 m^2^ (SD)	98.0 (11.1)	97.3 (12.6)	96.3 (13.3)	90.9 (19.6)	<0.001	97.3 (12.1)	97.2 (12.5)	95.8 (14.1)	92.3 (19.2)	<0.001

^a^Physical activity at Visit 1 was assessed using a leisure-time sprots index that ranged from 1 to 5. We assumed physcial activity scores were the same at Vist 1 and Visit 2. We reported physical activity scores with 2 decimal places to illustrate the trend more effectively.

^b^All diseases are prevalent diseases.

^c^*P*-values were calculated using chi-square tests for categorical variables and using ANOVA tests for continuous variables.

ARIC, Atherosclerosis Risk in Communities; BMI, body mass index; CVD, cardiovascular disease; eGFR, estimated glomerular filtration rate; HRT, hormone replacement therapy; PAC, proteomic aging clock; SD, standard deviation.

Among those 8,768 participants at Visit 2, 5,294 died by 2019 (1,734 died due to CVD, 1,516 died due to cancer, and 522 died due to LRD) with a mean follow-up of 21.42 years (SD = 8.17, range: 0.01 to 29.90 years). Age acceleration for the midlife ARIC PAC and published PACs showed associations of similar magnitude with all mortality types (Tables 3 and S8). For the midlife ARIC PAC, a one SD (SD = 2.94 years) increase in age acceleration was associated with a 38% increased risk of all-cause mortality [95% CI: 1.34, 1.42, *p* < 0.001], a 20% increased risk of CVD mortality [95% CI: 1.14, 1.27, *p* < 0.001], and a 36% increased risk of LRD mortality [95% CI: 1.22, 1.51, *p* < 0.001] (Table 3). Neither age acceleration for the midlife ARIC PAC nor published PACs was associated with cancer mortality (Table 3).

**Table 3:** The associations between age acceleration for the midlife ARIC PAC and Lehallier’s PAC and mortality; ARIC (1990–2019).

	No. of participants	No. of deaths	Total person-years	HR (95% CI)^a^ per one SD of age acceleration			
				Midlife ARIC PAC	*p*-value	Midlife Lehallier’s PAC	*p*-value
				(*SD = 2*.*94 years)*		(*SD = 3*.*00 years*)	
All-cause mortality	8,768	5,294	182,630	1.38 (1.34, 1.42)	<0.001	1.34 (1.30, 1.38)	<0.001
CVD mortality (Fine and Gray model)	8,768	1,734	182,630	1.20 (1.14, 1.27)	<0.001	1.19 (1.13, 1.25)	<0.001
Cancer mortality (Fine and Gray model)	8,768	1,516	182,630	1.04 (0.98, 1.10)	0.280	1.05 (0.99, 1.12)	0.260
LRD mortality (Fine and Gray model)	8,768	522	182,630	1.36 (1.22, 1.51)	<0.001	1.30 (1.17, 1.45)	<0.001

^a^ The model was adjusted for chronological age, sex, joint terms for race and study center (black participants from Mississippi; black participants from any other centers; white participants from Maryland; white participants from North Carolina; and white participants from Minnesota), education, BMI, smoking status, pack-years of smoking, alcohol intake, physical activity (at Visit 1), hormone replacement therapy, diabetes, hypertension, CVD, and eGFR at Visit 2.

ARIC, Atherosclerosis Risk in Communities; BMI, body mass index; CI, confidence interval; CVD, cardiovascular disease; eGFR, estimated glomerular filtration rate; HR, hazard ratio; LRD, lower respiratory disease; PAC, proteomic aging clock; SD, standard deviation.

### Late-life PACs

**Fig 4:**
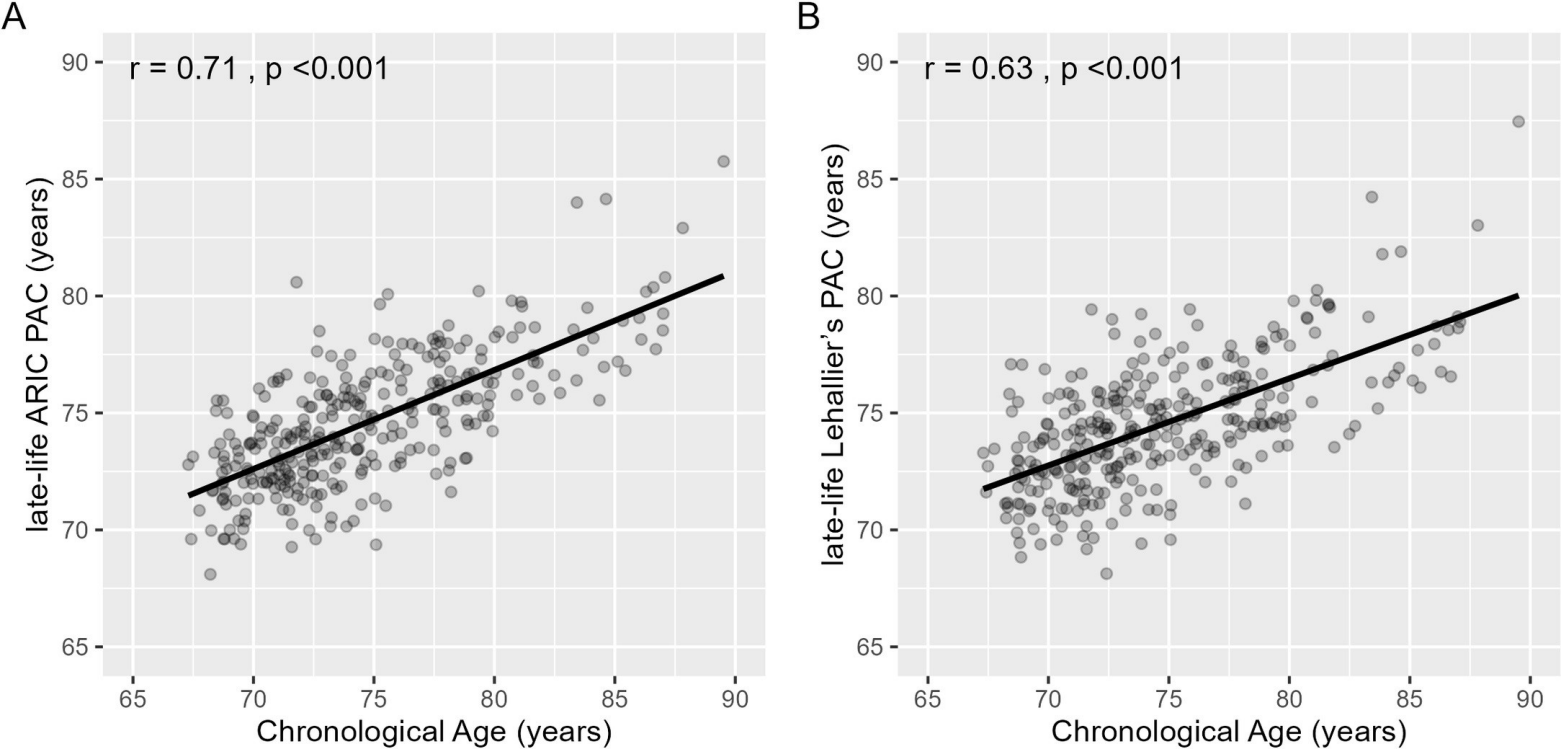
Pearson correlation (r) between the late-life ARIC PAC and Lehallier’s PAC and chronological age in the Visit 5 test set of healthy participants, ARIC. The x-axis depicts chronological age. The y-axis represents PAC. (A) The late-life ARIC PAC was constructed using healthy participants from ARIC. The correlation between the late-life ARIC PAC and chronological age was 0.71 (*p* < 0.001). (B) Lehallier’s PAC was computed using ARIC weights obtained from Ridge regression based on proteins available in ARIC. The correlation between late-life Lehallier’s PAC and chronological age was 0.63 (*p* < 0.001). ARIC, Atherosclerosis Risk in Communities; PAC, proteomic aging clock.

Elastic net regression selected 135 aptamers for the late-life ARIC PAC (Table 1), and 73% (*N* = 98) of these 135 aptamers were significantly associated (*p* < 0.050) with chronological age in a single protein model. In the Visit 5 test set, the late-life ARIC PAC was correlated with chronological age (r = 0.71, *p* < 0.001, MAE = 2.36 years, Table 1 and Fig 4A). The late-life Lehallier’s PAC had a correlation of 0.63 (*p* < 0.001) with chronological age (Table 1 and Fig 4B) and the late-life Tanaka’s and Sathyan’s PACs had correlations of 0.59 (*p* < 0.001) and 0.70 (*p* < 0.001) with chronological age, respectively (S6 Table and S2 Fig). In the Visit 5 test set, the late-life ARIC PAC was strongly correlated with the late-life Lehallier’s (r = 0.84, *p* < 0.001), Tanaka’s (r = 0.79, *p* < 0.001), and Sathyan’s PACs (r = 0.84, *p* < 0.001) (S9 Table).

After excluding the Visit 5 training set, the remaining 4,553 participants in late life were on average 76.5 ± 5.3 years old, 56.3% were female, and 19.7% self-identified as black. Distribution of late-life characteristics (Visit 5) across quartiles of late-life age acceleration are shown in Tables 4 and S10. Among the remaining participants in late life, the range of age acceleration was from −7.5 to +17.0 years for the late-life ARIC PAC. The distributions of characteristics including having a college-level education, physical activity, CVD, and eGFR were in the same direction across age acceleration for the late-life ARIC and published PACs (Tables 4 and S10). However, the percentages of white participants, never smokers, and never drinkers, and having hypertension and diabetes were different across different PACs (Tables 4 and S10).

**Table 4:** Visit 5 participants’ characteristics across quartiles of age acceleration for late-life ARIC PAC and Lehallier’s PAC; ARIC.

	**Late-life ARIC PAC**					**Late-life Lehallier’s PAC**				
	Q1 (*N* = 1,138)	Q2 (*N* = 1,138)	Q3 (*N* = 1,139)	Q4 (*N* = 1,138)	*P*-value^c^	Q1 (*N* = 1,138)	Q2 (*N* = 1,138)	Q3 (*N* = 1,139)	Q4 (*N* = 1,138)	*P*-value^c^
Age acceleration (min to max), years	−7.5 to −1.8	−1.7 to −0.2	−0.1 to +1.4	+1.5 to +17.0		−9.1 to −1.8	−1.7 to −0.2	−0.1 to +1.5	+1.6 to +14.4	
Mean age acceleration, years	−3.03	−0.97	0.59	3.40		−3.03	−0.93	0.62	3.34	
Chronological age, years (SD)	76.9 (5.1)	75.9 (5.0)	76.1 (5.3)	76.7 (5.4)	<0.001	76.7 (5.2)	76.3 (5.1)	76.0 (5.2)	76.8 (5.5)	<0.001
Female, %	57.7	57.5	57.1	52.9	0.061	62.2	59.8	53.9	49.5	<0.001
White, %	75.9	83.0	82.5	79.6	<0.001	78.6	80.5	81.9	80.1	0.251
Education, %										
<High school	13.7	11.7	15.2	17.0	0.012	11.3	13.3	14.8	18.2	<0.001
High school/vocational	41.3	44.4	42.1	42.6		41.7	42.8	42.7	43.2	
College	44.9	43.8	42.7	40.4		47.0	43.9	42.5	38.6	
BMI, kg/m^2^ (SD)	29.1 (4.9)	28.8 (5.2)	28.6 (5.7)	28.6 (6.7)	0.145	28.8 (5.0)	28.6 (5.3)	29.2 (6.2)	28.7 (6.1)	0.186
Smoking status, %										
Current smoker	4.0	5.1	6.8	10.0	<0.001	3.6	6.7	6.4	9.2	<0.001
Former smoker	54.5	56.8	50.1	50.8		54.4	50.3	54.1	53.6	
Never smoker	41.4	38.1	43.2	39.1		42.0	43.0	39.5	37.2	
Pack-years of smoking among ever smokers, pack-years (SD)	10.9 (16.3)	11.9 (19.0)	12.7 (21.8)	15.4 (22.3)	<0.001	10.5 (16.8)	12.1 (21.2)	12.2 (19.0)	16.0 (22.2)	<0.001
Alcohol intake, %										
Current drinker	53.2	50.6	49.1	46.1	0.009	53.0	50.6	49.0	46.6	0.118
Former drinker	28.3	30.8	28.4	31.0		28.7	29.0	29.7	31.2	
Never drinker	18.5	18.6	22.5	22.8		18.3	20.4	21.3	22.2	
Physical activity^a^, scores (SD)	2.70 (0.8)	2.64 (0.8)	2.56 (0.8)	2.39 (0.8)	<0.001	2.71 (0.8)	2.64 (0.8)	2.54 (0.8)	2.39 (0.8)	<0.001
Aspirin use in the preceding 2 weeks, %	68.8	69.8	71.0	73.0	0.141	69.0	69.7	71.3	72.6	0.234
Diabetes^b^, %	40.5	36.3	34.0	39.2	0.090	32.4	35.3	38.5	43.8	<0.001
Hypertension^b^, %	75.5	74.9	75.6	82.1	<0.001	72.7	76.2	77.0	82.2	<0.001
CVD^b^, %	19.9	24.7	29.8	38.4	<0.001	19.6	23.3	28.4	41.5	<0.001
eGFR, ml/min/1.73 m^2^ (SD)	77.9 (13.8)	73.6 (14.9)	70.6 (16.7)	59.9 (20.2)	<0.001	76.2 (14.3)	73.6 (15.9)	70.5 (16.8)	61.6 (20.3)	<0.001

^a^Physical activity was assessed using a leisure-time sprots index that ranged from 1 to 5. We reported physical activity scores with 2 decimal places to illustrate the trend more effectively.

^b^All the diseases are prevalent diseases.

^c^*P*-values were calculated using chi-square for categorical variables and using ANOVA for continuous variables.

ARIC, Atherosclerosis Risk in Communities; BMI, body mass index; CVD, cardiovascular disease; eGFR, estimated glomerular filtration rate; PAC, proteomic aging clock; SD, standard deviation.

Among those 4,553 participants at Visit 5, 1,123 died by 2019 (348 died due to CVD, 278 died due to cancer, and 128 died due to LRD) with a mean follow-up of 6.89 years (SD = 1.78, range: 0.07 to 8.58 years). Age acceleration for the late-life ARIC PAC and 3 published PACs were similarly associated with all mortality types (Tables 5 and S11). For the late-life ARIC PAC, a one SD (SD = 2.61 years) increase in age acceleration was associated with an increased risk of all-cause mortality [HR (95% CI) = 1.65 (1.52, 1.79), *p* < 0.001], CVD mortality [HR (95% CI) = 1.37 (1.18, 1.58), *p* < 0.001], cancer mortality [HR (95% CI) = 1.21 (1.02, 1.44), *p* = 0.028], and LRD mortality [HR (95% CI) = 1.68 (1.32, 2.12), *p* < 0.001] (Table 5).

**Table 5:** The associations of age acceleration for the late-life ARIC PAC and Lehallier’s PAC and the change in age acceleration from midlife to late life with mortality; ARIC (2011–2019).

	No. of participants	No. of deaths	Total person-years	HR (95% CI)^a^ per one SD of age acceleration			
				Late-life ARIC PAC (*SD = 2*.*61 years*)	*p*-value	Late-life Lehallier’s PAC (*SD = 2*.*54 years*)	*p*-value
All-cause mortality	4,553	1,123	29,356	1.65 (1.52, 1.79)	<0.001	1.58 (1.46, 1.72)	<0.001
CVD mortality (Fine and Gray model)	4,553	348	29,356	1.37 (1.18, 1.58)	<0.001	1.38 (1.19, 1.62)	<0.001
Cancer mortality (Fine and Gray model)	4,553	278	29,356	1.21 (1.02, 1.44)	0.028	1.19 (1.02, 1.40)	0.031
LRD mortality (Fine and Gray model)	4,553	128	29,356	1.68 (1.32, 2.12)	<0.001	1.57 (1.21, 2.03)	<0.001
	**The change in age acceleration from midlife to late life** ^b^						
	No. of participants	No. of deaths	Total person-years	HR (95% CI) per one SD of the change in age acceleration (*SD = 2*.*91 years*)	*p*-value		
All-cause mortality	2,707	736	17,081	1.71 (1.52, 1.94)	<0.001	NA^c^	
CVD mortality (Fine and Gray model)	2,707	239	17,081	1.38 (1.13, 1.68)	0.001		
Cancer mortality (Fine and Gray model)	2,707	172	17,081	1.30 (0.98, 1.71)	0.062		
LRD mortality (Fine and Gray model)	2,707	94	17,081	1.46 (1.05, 2.04)	0.025		

^a^The model was adjusted for chronological age, sex, joint terms for race and study center (black participants from Mississippi; black participants from any other centers; white participants from Maryland; white participants from North Carolina; and white participants from Minnesota), education, BMI, smoking status, pack-years of smoking, alcohol intake, physical activity, diabetes, hypertension, CVD, and eGFR at Visit 5.

^b^The associations for the change in age acceleration was examined among the 2,707 participants who survived until Visit 5 after excluding the training sets at Visit 2 and at Visit 5 and the model was additionally adjusted for midlife age acceleration.

^c^The associations between the change in age acceleration and mortality were examined using the ARIC PACs only because the ARIC PACs and published PACs showed similar associations with all mortality types.

ARIC, Atherosclerosis Risk in Communities; BMI, body mass index; CI, confidence interval; CVD, cardiovascular disease; eGFR, estimated glomerular filtration rate; HR, hazard ratio; LRD, lower respriatory disease; PAC, proteomic aging clock; SD, standard deviation.

### Associations of the change in age acceleration from midlife to late life with mortality

The median timespan between Visit 2 and Visit 5 was 20.8 years, ranging from 18.6 to 23.5 years. Among the 2,707 participants who survived up to Visit 5 (after excluding the training sets at Visit 2 and Visit 5), the midlife and late-life ARIC PACs were correlated with each other (r = 0.69, *p* < 0.001) and 48.4% of participants had a greater age acceleration in late life compared to midlife. Among those 2,707 participants, 736 died by 2019 (237 died due to CVD, 172 died due to cancer, and 94 died due to LRD) with a mean follow-up of 6.72 years (SD = 1.88, range: 0.07 to 8.58 years). In the fully adjusted model (additionally adjusted for midlife age acceleration), the change in age acceleration from midlife to late life was associated with all-cause mortality, CVD mortality, and LRD mortality, but not cancer mortality. HRs (95% CIs) per one SD of the change in age acceleration were 1.71 (1.52, 1.94), *p* < 0.001 for all-cause mortality, 1.38 (1.13, 1.68), *p* = 0.001 for CVD mortality, 1.46 (1.05, 2.04), *p* = 0.025 for LRD mortality, and 1.30 (0.98, 1.71), *p* = 0.062 for cancer mortality (Table 5). Midlife age acceleration did not modify the associations between the change in age acceleration and all-cause mortality (p-interaction for the multiplicative term = 0.264), CVD mortality (p-interaction = 0.644), LRD mortality (p-interaction = 0.256), or cancer mortality (p-interaction = 0.578).

### Association between midlife participants’ characteristics and late-life age acceleration

In the multivariable analysis of midlife participants’ characteristics (Visit 2), we found that being current smokers, never drinkers, or having diabetes, hypertension, CVD, a higher BMI, higher pack-years of smoking, lower eGFR or lower physical activity in midlife were associated with higher late-life age acceleration (Table 6).

**Table 6:** Association^a^ between midlife participants’ characteristics and late-life age acceleration, i.e., age acceleration for the late-life ARIC PAC; ARIC.

Midlife participants’ characteristics^b^	Coefficients	*P*-value or P-trend^c^
Chronological age	−0.03	<0.001
Male	0.05	0.652
Black	−0.67	<0.001
Education		
<High school	Ref (0)	0.267
High school/vocational	−0.21	
College	−0.16	
BMI	0.04	<0.001
Smoking status		
Never smoker	Ref (0)	<0.001
Former smoker	−0.25	
Current smoker	0.32	
Pack-years of smoking	0.01	<0.001
Alcohol intake		
Never drinker	Ref (0)	0.044
Former drinker	−0.14	
Current drinker	−0.27	
Physical activity	−0.08	0.131
Hormone replacement therapy		
Female never user	Ref (0)	0.043
Female ever user	−0.23	
Male	0	
Hypertension	0.45	<0.001
CVD	0.45	0.006
Diabetes	1.04	<0.001
eGFR	−0.03	<0.001

^a^The association was examined among the 4,553 participants who had information on the late-life ARIC PAC (after excluding the Visit 5 training set), and participants’ characteristics were included into model simultaneously.

^b^Physical activity at Visit 1 was assessed using a leisure-time sprots index that ranged from 1 to 5. We assumed that physical activity scores remained the same at Visit 1 and Visit 2. All the other characteristics were collected at Visit 2.

^c^*P*-value for continuous variables and P-trend for categorical variables.

ARIC, Atherosclerosis Risk in Communities; BMI, body mass index; CVD, cardiovascular disease; eGFR, estimated glomerular filtration rate; PAC, proteomic aging clock.

### Comparison of the associations between age acceleration and mortality in the full cohort and the cohort subset after excluding the training set

The magnitudes of associations of age acceleration for Sathyan’s PAC in both midlife and late life with mortality in all participants at each visit were comparable to the magnitudes of those associations in participants after excluding the training set (S12 Table). This results indicate that the exclusion of the training set did not influence the association between PACs and mortality.

### Proteins included in PACs

There are 49 common aptamers (with a non-zero weight) included in both the midlife and late-life ARIC PACs, accounting for 6.2% of all proteins in the midlife ARIC PAC and 36.4% of all proteins in the late-life ARIC PAC (S3 Fig). Nine proteins were found in common across the 3 published PACs and either the midlife or the late-life ARIC PACs: pleiotrophin (PTN), a disintegrin and metalloproteinase with thrombospondin motifs 5 (ADAMTS-5), macrophage metalloelastase (MMP12), cell adhesion molecule-related/down-regulated by oncogenes (CDON), growth differentiation factor 15 (GDF15), immunoglobulin superfamily containing leucine-rich repeat protein 2 (ISLR2), Kallikrein-7 (KLK7), Lactoperoxidase (LPO), and R-spondin-4 (RSPO4). These proteins have functions in inflammation, play a role in cell growth and survival, and are related to immune function (S13 Table).

We also identified 20 proteins in each ARIC PAC (midlife and late-life) based on the largest absolute weights of their constituting aptamers (S14 Table**)**. We found 6 proteins whose corresponding aptamers had the largest absolute weights in both ARIC PACs: transgelin (TAGL), WNT1-inducible-signaling pathway protein 2 (WISP-2), chordin-like protein 1 (CRDK1), collagen alpha-1(XV) chain (COF1), complement component C1q receptor (C1QR1), and pleiotrophin (PTN).

### Associations between age acceleration and mortality stratified by sex, race, and chronological age

The results for the associations between midlife PACs and mortality stratified by sex, race, and chronological age are presented in S15 Table and S4 Fig. Chronological age (in tertiles) statistically modified the associations of age acceleration for both the midlife ARIC PAC and 3 published PACs with CVD mortality (p-interactions ≤ 0.004), and the association was strongest among participants aged 47 to 54 years (first tertile) (S15 Table and S4 Fig).

The results for the associations between late-life PACs and mortality stratified by sex, race, and chronological age are presented in S16 Table and S5 Fig. Sex statistically modified the association between age acceleration and cancer mortality (p-interactions ≤ 0.038) for the late-life ARIC PAC as well as the 3 published PACs, and the association was stronger and significant in women for all PACs (S16 Table and S5 Fig). In addition, chronological age (in tertiles) significantly modified the association between age acceleration and CVD mortality (p-interaction = 0.036) for the late-life ARIC PAC but not the published PACs (S16 Table and S5 Fig).

### Association between age acceleration for the midlife ARIC PAC and 10-year risk of death

Among the 8,768 participants in midlife, a total of 1,137 participants died within 10 years, including 430 deaths attributed to CVD, 434 to cancer, and 85 to LRD (mean follow-up = 9.43 years, SD = 1.78, range: 0.01 to 10 years). In the fully adjusted model, a one SD (SD = 2.94 years) increase in age acceleration for the midlife ARIC PAC was associated with an increased risk of all-cause mortality [HR (95% CI) = 1.49 (1.41, 1.58), *p* < 0.001], CVD mortality [HR (95% CI) = 1.47 (1.33, 1.62), *p* < 0.001], cancer mortality [HR (95% CI) = 1.21 (1.09, 1.34), *p* < 0.001], and LRD mortality [HR (95% CI) = 1.95 (1.60, 2.38), *p* < 0.001].

### Application of the midlife ARIC PAC to late-life participants and application of the late-life ARIC PAC to midlife participants

When we applied the midlife ARIC PAC to late-life participants in ARIC, per one SD increase in age acceleration was associated with a 48% increase in the hazard of all-cause mortality [95% CI: 1.36, 1.63, *p* < 0.001], which was weaker compared to the association for the late-life ARIC PAC in late-life participants [1.65 (1.52, 1.79), *p* < 0.001]. When we applied the late-life ARIC PAC to midlife participants, per one SD increase in age acceleration was associated with a 40% increase in the hazard of all-cause mortality [95% CI: 1.36, 1.45, *p* < 0.001], which was comparable to the association for the midlife ARIC PAC in midlife participants [1.38 (1.34, 1.42), *p* < 0.001] but weaker compared to the association in late-life participants.

### External validation of the midlife ARIC PAC in the MESA study

Among 4,288 participants who were 46 to 70 years old and had proteomics data at Exam 1, 48.0% were female, and 38.5% self-identified as white, 26.9% as black, 23.0% as Hispanic, and 11.6% as Chinese. A total of 660 participants died by 2018 (155 died due to CVD, 239 died due to cancer, 49 died due to LRD) with a mean follow-up of 16.00 years (SD = 3.32, range: 0.17 to 18.43 years).

The correlation coefficient between the midlife ARIC PAC and chronological age was 0.83 (*p* < 0.001) at Exam 1. In the fully adjusted model, a one SD (SD = 2.86 years) increase in age acceleration for the midlife ARIC PAC was statistically significantly associated with all-cause mortality [HR (95% CI) = 1.45 (1.34, 1.56), *p* < 0.001] in MESA, similar to the association in ARIC. The association between age acceleration for the midlife ARIC PAC and all-cause mortality in MESA appeared to be similar in white [HR (95% CI) = 1.35 (1.17 to 1.56), *p* < 0.001] and black participants [1.36 (1.19, 1.56), *p* < 0.001] and those associations were similar to the associations with all-cause mortality in white [1.36 (1.31, 1.41), *p* < 0.001] and black participants [1.40 (1.32, 1.50), *p* < 0.001] in ARIC. However, in MESA, the associations with all-cause mortality in white and black participants were weaker than the associations in Chinese [1.74 (1.28, 2.38), *p* < 0.001] and Hispanic participants [1.83 (1.57, 2.13), *p* < 0.001]. One SD increase in age acceleration for the midlife ARIC PAC was also associated with CVD mortality [HR (95% CI) = 1.45 (1.22, 1.72), *p* < 0.001], and LRD mortality [HR (95% CI) = 1.56 (1.26, 1.93), *p* < 0.001], but not cancer mortality [HR (95% CI) = 1.12 (0.97, 1.27), *p* = 0.149] in MESA, similar to the association for the midlife ARIC PAC in ARIC. In MESA Exam 1, Lehallier’s PAC was correlated with chronological age (r = 0.79, *p* < 0.001). One SD increase in age acceleration for Lehallier’s PAC was associated with all-cause mortality [HR (95% CI) = 1.32 (1.22, 1.42), *p* < 0.001], CVD mortality [1.43 (1.19, 1.72), *p* < 0.001], and LRD mortality [1.47 (1.16, 1.87), *p* < 0.001], but not cancer mortality [1.03 (0.89, 1.18), *p* = 0.730]. The associations between Lehallier’s PAC and mortality in MESA were similar to the associations for the midlife ARIC PAC in MESA.

## Discussion

In a large prospective community-based study of white and black individuals, the ARIC study, we tested 3 published PACs [5,6,19] and constructed and validated de novo PACs in midlife (46 to 70 years) and late life (66 to 90 years), using 4,955 aptamers measured by the SomaScan assay (v.4). Both the midlife and late-life ARIC PACs were developed in healthy participants and were strongly correlated with chronological age. Correlations between chronological age and the ARIC PACs were 0.80 in midlife and 0.71 in late life, which were slightly stronger compared to the correlations between chronological age and the 3 published PACs (Lehallier’s, Tanaka’s, and Sathyan’s), respectively (r = 0.58 to 0.76 in midlife and r = 0.59 to 0.70 in late life). All the HRs for the associations with mortality, including mortality from all-cause, CVD, cancer, and LRD, were very similar for the ARIC and published PACs in midlife and late life, respectively. Notably, the associations with all-cause mortality, CVD mortality, and LRD mortality were significant at each visit but stronger in late life than in midlife, and the associations with cancer mortality were significant in late life only. The change in age acceleration from midlife to late life had associations of similar magnitude with all-cause mortality and CVD mortality when compared to the associations for the late-life ARIC PAC. The HR estimate for LRD mortality was slightly lower for the change in age acceleration compared to the late-life ARIC PAC, but the confidence intervals for these 2 estimates largely overlapped. The change in age acceleration was not associated with cancer mortality. The external validation of the midlife ARIC PAC in the MESA study showed high correlation with chronological age. In addition, the midlife ARIC PAC showed significant associations with mortality, as observed in ARIC midlife participants.

In midlife, we applied different penalized regressions and various transformations of proteins to develop 5 de novo ARIC PACs, including a PAC that accounted for nonlinear associations between proteins and chronological age. These 5 PACs were highly correlated with each other. Thus, among these 5 PACs, we selected the midlife ARIC PAC, constructed using the simplest protein transformation, i.e., log2-transformed without any further transformation. We selected the PAC with the simplest protein transformation because, if validated, it would be easier to use this PAC in future studies. We also constructed the late-life ARIC PAC using the same method as employed for the midlife ARIC PAC. In our study, elastic net regression selected 788 aptamers for the midlife ARIC PAC and 135 aptamers for the late-life ARIC PAC. The smaller number of aptamers for the late-life ARIC PAC may be because of the smaller training set at Visit 5 (*N* = 630) compared to the Visit 2 training set (*N* = 2,993). A larger training set allows penalized regressions to select more proteins, including those with a modest association with age. This is in agreement with Sathyan’s PAC of 162 proteins, which was developed using the same SomaScan assay as in our study with a training set of 500 participants [19].

We compared associations of the midlife and late-life ARIC PACs and published PACs with mortality. Although different PACs included different proteins, age acceleration for both the ARIC PACs and published PACs showed comparable associations with mortality at each time point. Our findings for all-cause mortality in midlife participants were similar to the findings in the InCHIANTI study (*N* = 459, chronological age: 21 to 98 years) by Tanaka and colleagues. In their study, they reported a significant association between age acceleration for Tanaka’s PAC and all-cause mortality after adjusting for chronological age, sex, and study site [HR (95% CI) per 1 SD = 1.29 (1.11 to 1.50)] [22]. Since the published PACs and our ARIC PACs consist of different number of proteins but are similarly associated with mortality, it appears that not the number of proteins drive the performance of PACs.

Lehallier’s PAC and Sathyan’s PAC were developed in primarily white individuals, while the ARIC PACs were developed in a cohort of white and black participants. Although proteins associated with age varied by race [23–25], the use of a biracial population as the training set did not improve the performance of PACs, i.e., the ARIC PACs and published PACs showed associations of similar magnitude with mortality in the ARIC study. Moreover, in ARIC, race (white/black) did not modify the association between age acceleration and mortality. In parallel, in MESA, the association between age acceleration and mortality appeared to be similar among white and black participants and mirrored those in ARIC, but those associations were weaker compared to the associations with mortality in Chinese and Hispanic participants. However, the sample sizes for Chinese (58 deaths occurred by 2018) and Hispanic participants (145 deaths occurred) are limited in MESA. Future studies that include a large population from other racial/ethnic groups would help to understand whether our ARIC PACs could be applied to other racial/ethnic groups to predict mortality.

In our study, for both the ARIC PACs and published PACs, their midlife age acceleration showed weaker associations with all mortality types than late-life age acceleration. Moreover, when applying the midlife ARIC PAC to late-life participants, the association with mortality for midlife ARIC PAC was weaker compared to the association for the late-life ARIC PAC in late-life participants. These may be because midlife PACs were constructed using blood samples collected at midlife and failed to capture all the changes in aging that happen after midlife. The weaker association with mortality for midlife age acceleration may also explained by the potential regression dilution bias due to the longer follow-up of up to 29.9 years since midlife [44]. This is also supported by the results from our analyses: (1) when the follow-up was restricted to 10 years for midlife participants, the estimates for association with mortality became much stronger; and (2) when applying the late-life ARIC PAC to midlife participants, the association with mortality for late-life ARIC PAC in midlife participants was weaker compared to the association in late-life participants. It appears that both the panel of proteins in PACs and the length of follow-up may influence the associations between PACs and mortality.

Tanaka’s PAC was developed in 120 participants aged 22 to 93 years while Sathyan’s PAC was developed in 500 participants aged 65 to 95 years. We found that Tanaka’s PAC, Sathyan’s PAC, and the ARIC PAC showed similar and significant associations with mortality in ARIC. However, the correlations of Tanaka’s PAC and Sathyan’s PAC with chronological age had different patterns in midlife and late-life participants. In ARIC, Tanaka’s PAC was similarly correlated with age in midlife participants (within the age range of Tanaka’s population) (r = 0.66) and late-life participants (within the age range of Tanaka’s population) (r = 0.59), but these correlations were much lower compared to the correlation of 0.94 with age reported in their original paper [5]. In ARIC, Sathyan’s PAC had a higher correlation with age in late-life participants (within the age range of Sathyan’s population) (r = 0.70) compared to the correlation in midlife participants (outside the age range of Sathyan’s population) (r = 0.58). The correlation in late-life participants in ARIC for Sathyan’s PAC was a little bit lower compared to the correlation of 0.79 reported in their original paper [19]. These findings suggest that it may be slightly better to apply PACs to individuals within the age range of the population used to develop them, but they may also work when applied to individuals outside the age range.

Our findings showed that midlife individuals who were current smokers (compared to never smokers), as well as those with higher (versus lower) BMI, lower (versus higher) eGFR, and age-related diseases, such as CVD, hypertension, and diabetes in midlife, were associated with higher age acceleration in late life. This is important given the modifiability and preventability of these factors, which may inform public health policies that aim to decrease the aging process. In addition, a larger change in age acceleration from midlife to late life was associated with an increased risk of all-cause mortality, CVD mortality, and LRD mortality. Future studies should incorporate multiple time points in applying PACs to model the change in age acceleration over time.

The strengths of this study include that the ARIC cohort includes a diverse sample comprising both white and black individuals, while previous studies of PACs either had small sample sizes or included mainly white individuals [5,6,19]. Also, we compared multiple PACs regarding their correlation with chronological age and their associations with mortality and validate the ARIC PAC by examining its association with mortality using the MESA study, an external cohort that differed in characteristics from ARIC. In addition, with the availability of proteomics data from 2 distinct visits (20 years apart) in ARIC, we were able to examine the association between the midlife to late-life change in age acceleration and mortality. Moreover, we adjusted for a broader range of confounders while previous studies of PACs only adjusted for demographic factors [19,22]. Our study has several possible limitations. First, the possibility of protein degradation during long-term storage cannot be excluded. However, in ARIC, the blood samples were frozen right after their collection, stored under controlled conditions, and have never been thawed, reducing the possibility of degradation. Furthermore, a previous ARIC study of proteomics demonstrated a similar coefficient of variation for proteomics in blood samples collected at Visit 2 (1990 to 1992, stored for a longer time) and at Visit 5 (2011 to 2013, stored for a shorter time) (CVBA = 6% at Visit 2 and 7% at Visit 5), suggesting no evidence of severe protein degradation [16,45–47]. Second, ARIC measured proteins in plasma, rather than other tissues, which limited the generalizability of our PACs to proteins from other tissues. Third, our ARIC PACs were constructed in midlife and late-life participants. It is not known whether our PACs can be applied to young individuals.

In the United States, the average human life expectancy has increased by 30 years during the 20th century. This increased life expectancy has given rise to the number of individuals living with age-related diseases and disabilities, which in turn lead to reduced health span, lower quality of life, and increased healthcare costs in the United States. Our results underscored the potential of PACs to serve as a blood biomarker for biological age. As the aging process and age-related diseases share the same basic molecular mechanisms, it is expected that targeting the aging process would reduce mortality and slow down the development of several age-related diseases simultaneously, potentially prolong health span. Currently, no gold standard measures of biological age exist; thus, there is a need in new metrics for measuring biological age, such as PACs. PACs could be used to predict age-related diseases, determine modifiable behavioral factors that drive accelerated aging and create disease-specific risk calculators [48]. In the future, PACs could help to identify optimal candidates for existing and emerging anti-aging interventions including lifestyle and therapeutic interventions that are currently in clinical trials or under development (including senolytics and senomorphics) [49]. Also, PACs could be applied as surrogate endpoints in clinical trials that study the effect of anti-aging interventions [50]; using PACs instead of real outcomes would substantially decrease the length and cost of those trials. Findings from those trials may help identify the best anti-aging interventions and inform physicians’s treatment decisions, as well as develop public health policies that aim to reduce the aging process and development of age-related diseases, potentially promoting health span.

In conclusion, we developed de novo midlife and late-life PACs in a diverse population of white and black individuals and showed that these PACs were associated with mortality risk. The magnitude of these associations is similar to the associations observed for previously published PACs, both in midlife and late life. Moreover, the change in age acceleration from midlife to late life showed comparable associations with mortality as the late-life PAC. The external validation of the midlife PAC showed significant associations with mortality. Future studies are recommended to investigate the potential use of PACs as biomarkers for biological age and risk stratification for age-related disease.

## Supporting information

S1 Appendix Assessment of diseases and characteristics of interests, as well as procedures for identifying healthy participants. (DOCX)

S2 Appendix Formulas (intercept and weights for proteins) for the ARIC and Published PACs. (XLSX)

S1 Fig Pearson correlation (r) between midlife Tanaka’s and Sathyan’s proteomic aging clocks (PACs) and chronological age in healthy participants of the Visit 2 test set, ARIC. The x-axis depicts chronological age. The y-axis represents proteomic aging clock (PAC). (A) Tanaka’s PAC was computed using ARIC weights obtained from Ridge regression based on proteins available in ARIC. The correlation between midlife Tanaka’s PAC and chronological age was 0.66 (*p* < 0.001). (B) Sathyan’s PAC was calculated using the published weights. The correlation between midlife Sathyan’s PAC and chronological age was 0.58 (*p* < 0.001).
(TIF)

S2 Fig Pearson correlation (r) between late-life Tanaka’s and Sathyan’s proteomic aging clocks (PACs) and chronological age in healthy participants of the Visit 5 test set, ARIC. The x-axis depicts chronological age. The y-axis represents proteomic aging clock (PAC). (A) Tanaka’s PAC was computed using ARIC weights obtained from Ridge regression based on proteins available in ARIC. The correlation between late-life Tanaka’s PAC and chronological age was 0.59 (*p* < 0.001). (B) Sathyan’s PAC was calculated using the published weights. The correlation between late-life Sathyan’s PAC and chronological age was 0.70 (*p* < 0.001).
(TIF)

S3 Fig Overlap of aptamers included in the midlife and late-life ARIC proteomic aging clocks (PACs). The gray circle shows the aptamers included in the midlife ARIC PAC and the yellow circle shows the aptamers included in the late-life ARIC PAC.
(TIF)

S4 Fig Association between age acceleration for the midlife ARIC and published PACs and mortality stratified by race, sex, and chronological age (in tertiles); ARIC (1990–2019). The plot shows the hazard ratio (HR) and 95% confidence interval (CI) for mortality with per one standard deviation (SD) increase in age acceleration for midlife ARIC PAC (A), midlife Lehallier’s PAC (B), midlife Tanaka’s PAC (C), and midlife Sathyan’s PAC (D).
(TIF)

S5 Fig Association between age acceleration for the late-life ARIC and published PACs and mortality stratified by race, sex, and chronological age (in tertiles); ARIC (2011–2019). The plot shows the hazard ratio (HR) and 95% confidence interval (CI) for mortality with per one standard deviation (SD) increase in age acceleration for late-life ARIC PAC (A), late-life Lehallier’s PAC (B), late-life Tanaka’s PAC (C), and late-life Sathyan’s PAC (D).
(TIF)

S1 Table Description of Lehallier’s, Tanaka’s, and Sathyan’s proteomic aging clocks (PACs). (DOCX)

S2 Table Description of the de novo ARIC proteomic aging clocks (PACs) constructed in midlife healthy participants; ARIC. (DOCX)

S3 Table Pearson correlation coefficients between the de novo ARIC proteomic aging clocks (PACs) constructed in midlife healthy participants and published PACs among the Visit 2 test set of healthy participants. (DOCX)

S4 Table Including/excluding participants with controlled hypertension^a^ for late-life healthy participants at Visit 5 to construct proteomic aging clocks (PACs) using elastic net regression. (DOCX)

S5 Table R squared after regressing age acceleration for the midlife and late-life ARIC proteomic aging clocks (PACs) on covariates at the corresponding visits. (DOCX)

S6 Table Pearson correlation between Tanaka’s and Sathyan’s proteomic aging clocks (PACs) and chronological age and median absolute error (MAE); ARIC. (DOCX)

S7 Table Visit 2 participants’ characteristics across quartiles of age acceleration for midlife Tanaka’s and Sathyan’s PACs; ARIC. (DOCX)

S8 Table The association between age acceleration for midlife Tanaka’s and Sathyan’s PACs and mortality; ARIC (1990–2019). (DOCX)

S9 Table Pearson correlation coefficients between the late-life ARIC PAC and published PACs in the Visit 5 test set of healthy participants. (DOCX)

S10 Table Visit 5 participants’ characteristics across quartiles of age acceleration for late-life Tanaka’s and Sathyan’s PACs; ARIC. (DOCX)

S11 Table The associations of age acceleration for late-life Tanaka’s and Sathyan’s PACs with mortality; ARIC (2011–2019). (DOCX)

S12 Table The associations of age acceleration for Sathyan’s PAC with mortality in all ARIC participants. (DOCX)

S13 Table Common proteins included in published PACs and either the midlife or the late-life ARIC PACs. (DOCX)

S14 Table Top 20 proteins with the largest absolute weight in the midlife and late-life ARIC PACs. (DOCX)

S15 Table The association between age acceleration for the midlife ARIC PAC and published PACs and mortality stratified by sex, race, and chronological age (in tertiles); ARIC (1990–2019). (DOCX)

S16 Table The associations of age acceleration for the late-life ARIC PAC and published PACs with mortality stratified by sex, race, and chronological age (in tertiles); ARIC (2011–2019). (DOCX)

## Abbreviations

ARIC: Atherosclerosis Risk in Communities
BMI: body mass index
CI: confidence interval
COPD: chronic obstructive pulmonary disease
CVD: cardiovascular disease
eGFR: estimated glomerular filtration rate
HR: hazard ratio
HRT: hormone replacement therapy
ICD: International Classification of Diseases
LRD: lower respiratory disease
MAE: median absolute error
MESA: Multi-Ethnic Study of Atherosclerosis
NDI: National Death Index
RFU: relative fluorescent unit
SD: standard deviation
