# Supplementary material for: Development, characterization, and replication of proteomic aging clocks: Analysis of 2 population-based cohorts
Source: PLoS Med. 2024 Sep 24;21(9):e1004464. doi: 10.1371/journal.pmed.1004464 (PMC11460707; doi:10.1371/journal.pmed.1004464)
Supplement: S1 Appendix — (DOCX) [file pmed.1004464.s001.docx]

**S1 Appendix**

The assessment of cancer, diabetes, hypertension, abnormal kidney function, chronic obstructive pulmonary disease (COPD), cardiovascular disease (CVD) events, and other characteristics of interest, as well as the procedures for identifying healthy participants, are described below.

Ascertainment of cancer cases

Prevalent cancers at Visit 1 were defined as self-reported previous diagnoses of cancer. Incident cancer cases developed after Visit 1 were ascertained through 2015 via linkage with state cancer registries in Minnesota, North Carolina, Maryland, and Mississippi. Records were supplemented by abstracting medical records and hospital discharge codes (1).

Ascertainment of diabetes, hypertension, and abnormal kidney function

Diabetes mellitus at each visit was defined as fasting glucose ≥126 mg/dL, non-fasting glucose ≥200 mg/dL, treatment for diabetes mellitus, or self-reported physician diagnosis of diabetes.

Hypertension at each visit was defined as diastolic blood pressure ≥90 mmHg, systolic blood pressure ≥140 mmHg, or taking medication for high blood pressure. Hypertension is controlled if the measured diastolic blood pressure is below 90 and the measured systolic blood pressure is below 140 while the participant is on medication (2).

Abnormal kidney function was defined as an estimated glomerular filtration rate (eGFR) less than 60 mL/min/1.73m^2^. The eGFR at each visit was calculated based on serum creatinine and incorporated participants’ age and sex (3).

Ascertainment of COPD events

COPD was defined as having a forced expiratory volume in 1 second (FEV_1_) less than 80% of the predicted value and an FEV_1_/forced vital capacity (FVC) ratio below 0.7. Only Participants meeting both criteria were categorized as having COPD for analysis. The description of FEV_1_ and FVC measures in ARIC has been described previously (4).

Ascertainment of CVD events

CVD events consist of the following: 1) heart failure (HF), 2) definite or probable stroke, or 3) coronary heart disease (CHD), defined as definite or probable myocardial infarction (MI) or definite fatal CHD (10, 11). The definitions of pre-existing HF, CHD, and MI at Visit 1, as well as incident events that developed after Visit 1 in ARIC, have been previously described (10-16).

Assessment of other characteristics of interest

At each visit, each participant reported information on smoking status, alcohol intake, and medication use, and underwent a physical exam that included height and weight. Body mass index (BMI) was calculated as weight (kg) divided by height (in meters) squared. The calculation of pack-years of smoking at Visit 2 was previously described (17). Pack-years of smoking at Visit 5 were calculated by combining the pack-year estimates from Visit 4 with the smoking status estimates from Visit 4 because smoking status estimates were not available at Visit 5. Physical activity at Visit 1 and Visit 5 was assessed using a modified version of the Baecke questionnaire, from which leisure time physical activity was derived based on reported intensity and time spent on sports and exercise.

Identifying healthy participants at Visit 2 (midlife)

To identify the group of healthy participants at Visit 2, among the 11,761 White and Black participants with protein data available, we excluded those who reported a history of cancer (N = 927), participants who reported a diagnosis of diabetes (N = 1,857), participants who reported hypertension (N = 3,311), participants with abnormal kidney lab measurements (N = 43), participants with COPD (N = 841), and those who reported a history of CVD (N = 293), resulting 4,489 healthy participants at Visit 2.

Identifying healthy participants at Visit 5 (late life)

To identify the group of healthy participants at Visit 5, among the 5,183 White and Black participants with protein data available, we excluded participants who reported a history of cancer (N = 1,211), participants who reported a diagnosis of diabetes (N = 1,247), participants who had uncontrolled hypertension (N = 1,595), participants with abnormal kidney lab measurements (N = 385), participants with COPD (N = 537), and those who reported a history of CVD (N = 154), resulting in 945 healthy participants at Visit 5.

**References**

1. Joshu CE, Barber JR, Coresh J, Couper DJ, Mosley TH, Vitolins MZ, et al. Enhancing the Infrastructure of the Atherosclerosis Risk in Communities (ARIC) Study for Cancer Epidemiology Research: ARIC Cancer. Cancer Epidemiol Biomarkers Prev. 2018;27(3):295-305.

2. Lu J, Lu Y, Krumholz HM, Jiang L. Prevalence and control of hypertension - Authors' reply. Lancet. 2018;392(10155):1306.

3. Inker LA, Eneanya ND, Coresh J, Tighiouart H, Wang D, Sang Y, et al. New Creatinine- and Cystatin C-Based Equations to Estimate GFR without Race. N Engl J Med. 2021;385(19):1737-49.

4. Chamberlain AM, Schabath MB, Folsom AR. Associations of chronic obstructive pulmonary disease with all-cause mortality in Blacks and Whites: the atherosclerosis risk in communities (ARIC) study. Ethn Dis. 2009;19(3):308-14.

5. ATS statement--Snowbird workshop on standardization of spirometry. Am Rev Respir Dis. 1979;119(5):831-8.

6. Crapo RO, Morris AH, Gardner RM. Reference spirometric values using techniques and equipment that meet ATS recommendations. Am Rev Respir Dis. 1981;123(6):659-64.

7. Harik-Khan RI, Fleg JL, Muller DC, Wise RA. The effect of anthropometric and socioeconomic factors on the racial difference in lung function. Am J Respir Crit Care Med. 2001;164(9):1647-54.

8. Lung function testing: selection of reference values and interpretative strategies. American Thoracic Society. Am Rev Respir Dis. 1991;144(5):1202-18.

9. Glindmeyer HW, Lefante JJ, McColloster C, Jones RN, Weill H. Blue-collar normative spirometric values for Caucasian and African-American men and women aged 18 to 65. Am J Respir Crit Care Med. 1995;151(2 Pt 1):412-22.

10. Bell EJ, Lutsey PL, Windham BG, Folsom AR. Physical activity and cardiovascular disease in African Americans in Atherosclerosis Risk in Communities. Med Sci Sports Exerc. 2013;45(5):901-7.

11. Folsom AR, Yatsuya H, Nettleton JA, Lutsey PL, Cushman M, Rosamond WD, et al. Community prevalence of ideal cardiovascular health, by the American Heart Association definition, and relationship with cardiovascular disease incidence. J Am Coll Cardiol. 2011;57(16):1690-6.

12. Eriksson H, Caidahl K, Larsson B, Ohlson LO, Welin L, Wilhelmsen L, et al. Cardiac and pulmonary causes of dyspnoea--validation of a scoring test for clinical-epidemiological use: the Study of Men Born in 1913. Eur Heart J. 1987;8(9):1007-14.

13. Loehr LR, Rosamond WD, Chang PP, Folsom AR, Chambless LE. Heart failure incidence and survival (from the Atherosclerosis Risk in Communities study). Am J Cardiol. 2008;101(7):1016-22.

14. Rosamond WD, Chang PP, Baggett C, Johnson A, Bertoni AG, Shahar E, et al. Classification of heart failure in the atherosclerosis risk in communities (ARIC) study: a comparison of diagnostic criteria. Circ Heart Fail. 2012;5(2):152-9.

15. The National Survey of Stroke. National Institute of Neurological and Communicative Disorders and Stroke. Stroke. 1981;12(2 Pt 2 Suppl 1):I1-91.

16. Rosamond WD, Folsom AR, Chambless LE, Wang CH, McGovern PG, Howard G, et al. Stroke incidence and survival among middle-aged adults: 9-year follow-up of the Atherosclerosis Risk in Communities (ARIC) cohort. Stroke. 1999;30(4):736-43.

17. Polter EJ, Onyeaghala G, Lutsey PL, Folsom AR, Joshu CE, Platz EA, et al. Prospective Association of Serum and Dietary Magnesium with Colorectal Cancer Incidence. Cancer Epidemiol Biomarkers Prev. 2019;28(8):1292-9.

18. Lehallier B, Shokhirev MN, Wyss-Coray T, Johnson AA. Data mining of human plasma proteins generates a multitude of highly predictive aging clocks that reflect different aspects of aging. Aging Cell. 2020;19(11):e13256.

19. Tanaka T, Biancotto A, Moaddel R, Moore AZ, Gonzalez-Freire M, Aon MA, et al. Plasma proteomic signature of age in healthy humans. Aging Cell. 2018;17(5):e12799.

20. Sathyan S, Ayers E, Gao T, Weiss EF, Milman S, Verghese J, et al. Plasma proteomic profile of age, health span, and all-cause mortality in older adults. Aging Cell. 2020;19(11):e13250.
