# Supplementary figures and images for: Development, characterization, and replication of proteomic aging clocks: Analysis of 2 population-based cohorts
Source: PLoS Med. 2024 Sep 24;21(9):e1004464. doi: 10.1371/journal.pmed.1004464 (PMC11460707; doi:10.1371/journal.pmed.1004464)

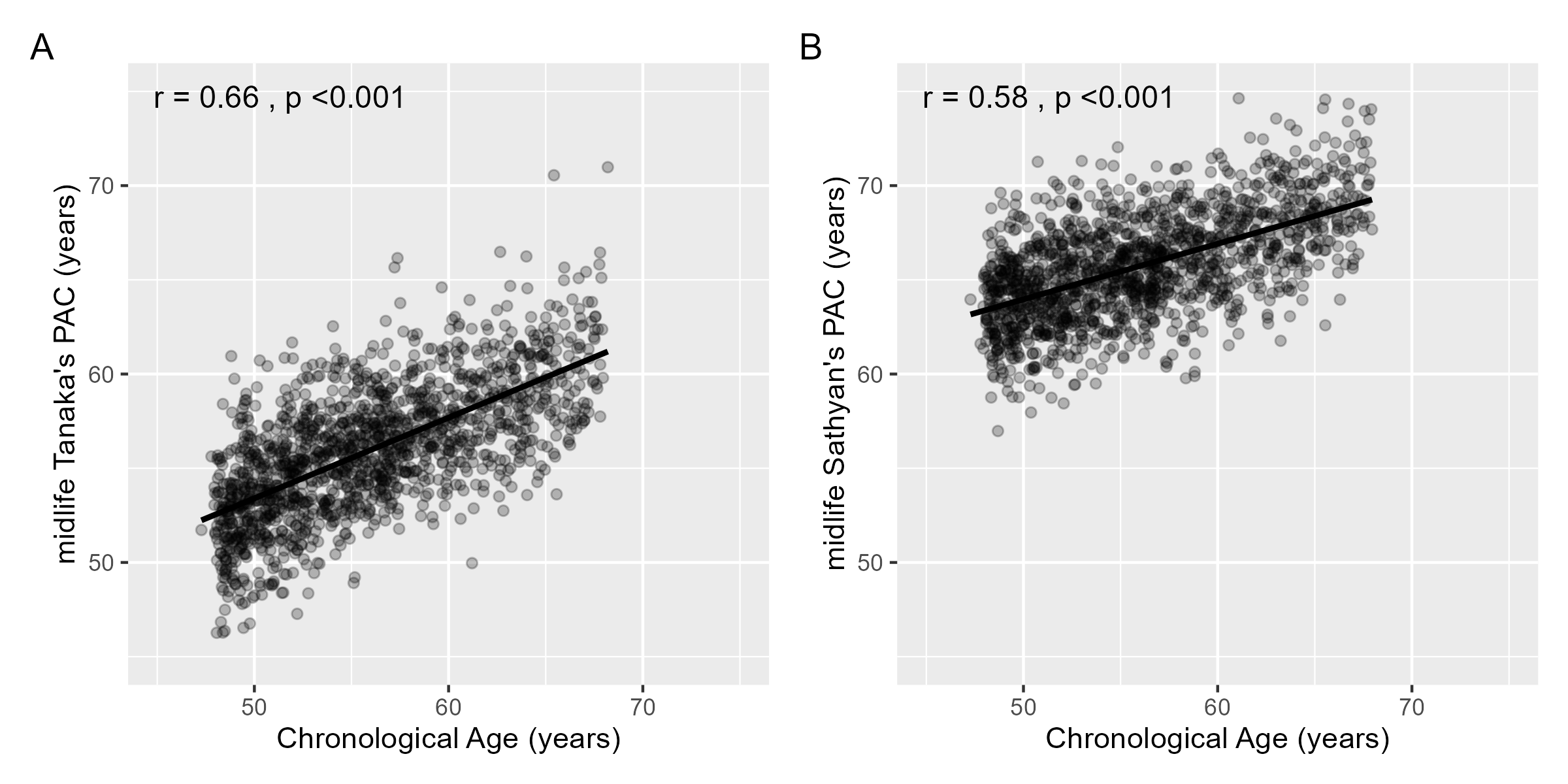

Supplement: S1 Fig — The x-axis depicts chronological age. The y-axis represents proteomic aging clock (PAC). (A) Tanaka’s PAC was computed using ARIC weights obtained from Ridge regression based on proteins available in ARIC. The correlation between midlife Tanaka’s PAC and chronological age was 0.66 (p < 0.001). (B) Sathyan’s PAC was calculated using the published weights. The correlation between midlife Sathyan’s PAC and chronological age was 0.58 (p < 0.001). (TIF) [file pmed.1004464.s003.tif]

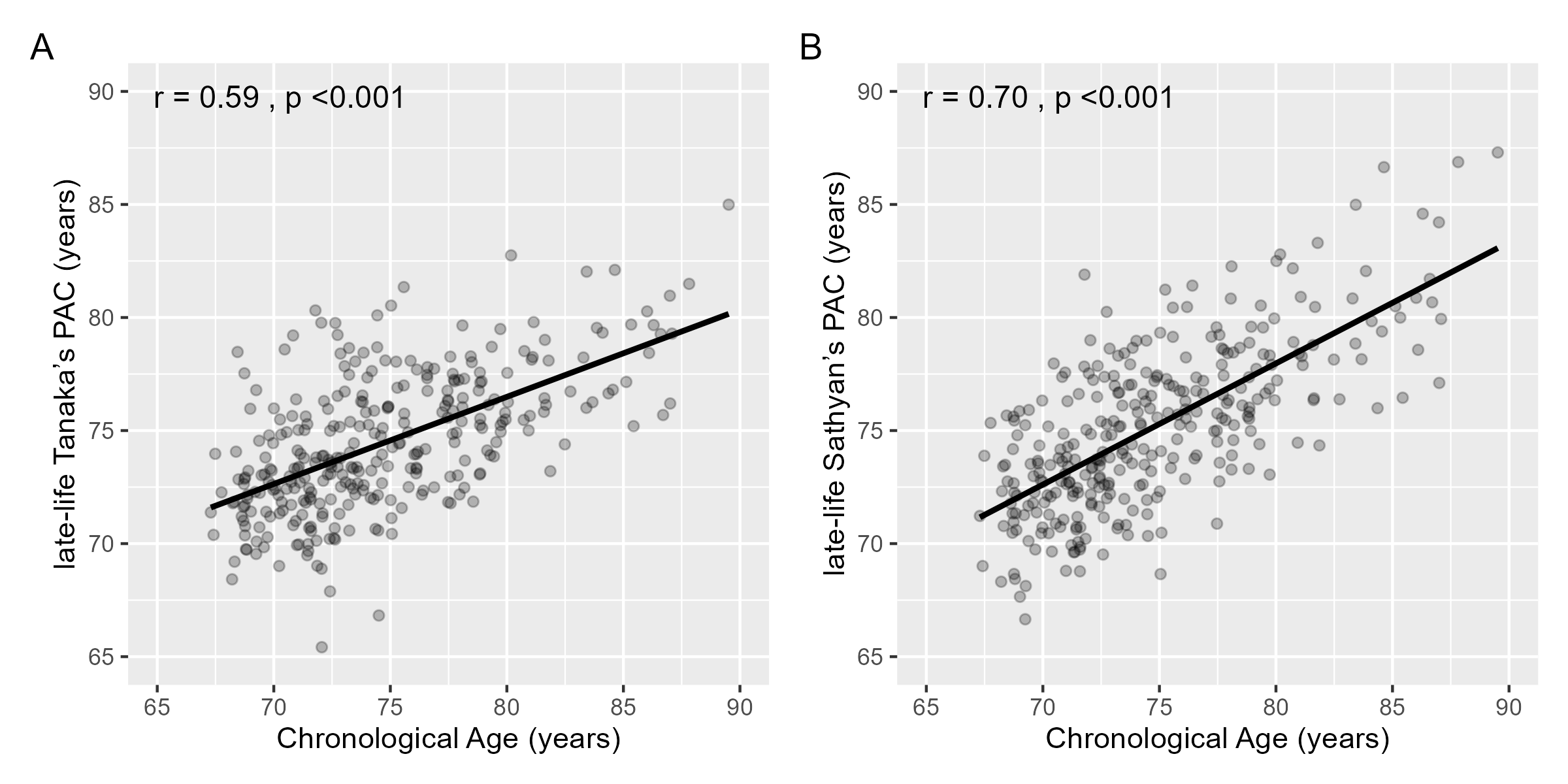

Supplement: S2 Fig — The x-axis depicts chronological age. The y-axis represents proteomic aging clock (PAC). (A) Tanaka’s PAC was computed using ARIC weights obtained from Ridge regression based on proteins available in ARIC. The correlation between late-life Tanaka’s PAC and chronological age was 0.59 (p < 0.001). (B) Sathyan’s PAC was calculated using the published weights. The correlation between late-life Sathyan’s PAC and chronological age was 0.70 (p < 0.001). (TIF) [file pmed.1004464.s004.tif]

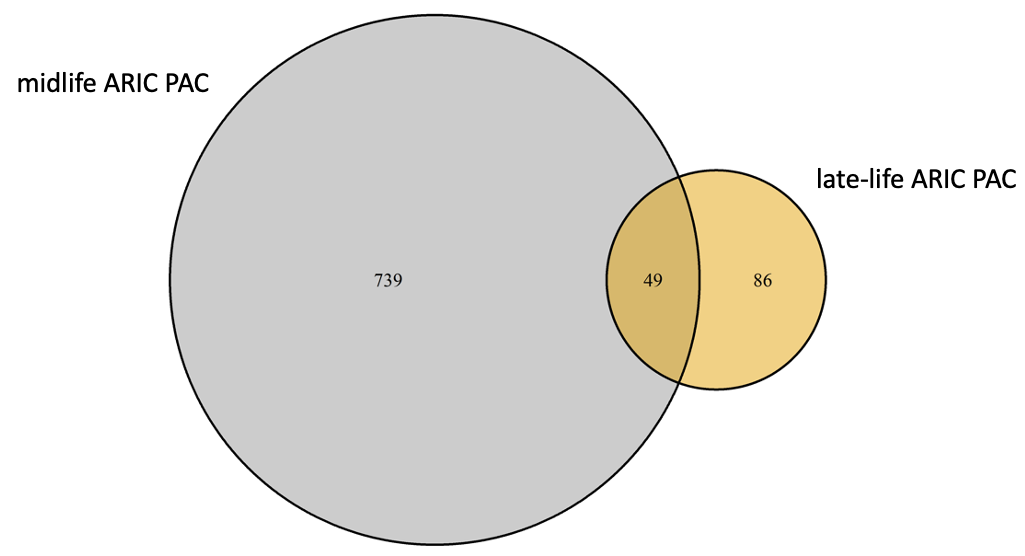

Supplement: S3 Fig — The gray circle shows the aptamers included in the midlife ARIC PAC and the yellow circle shows the aptamers included in the late-life ARIC PAC. (TIF) [file pmed.1004464.s005.tif]

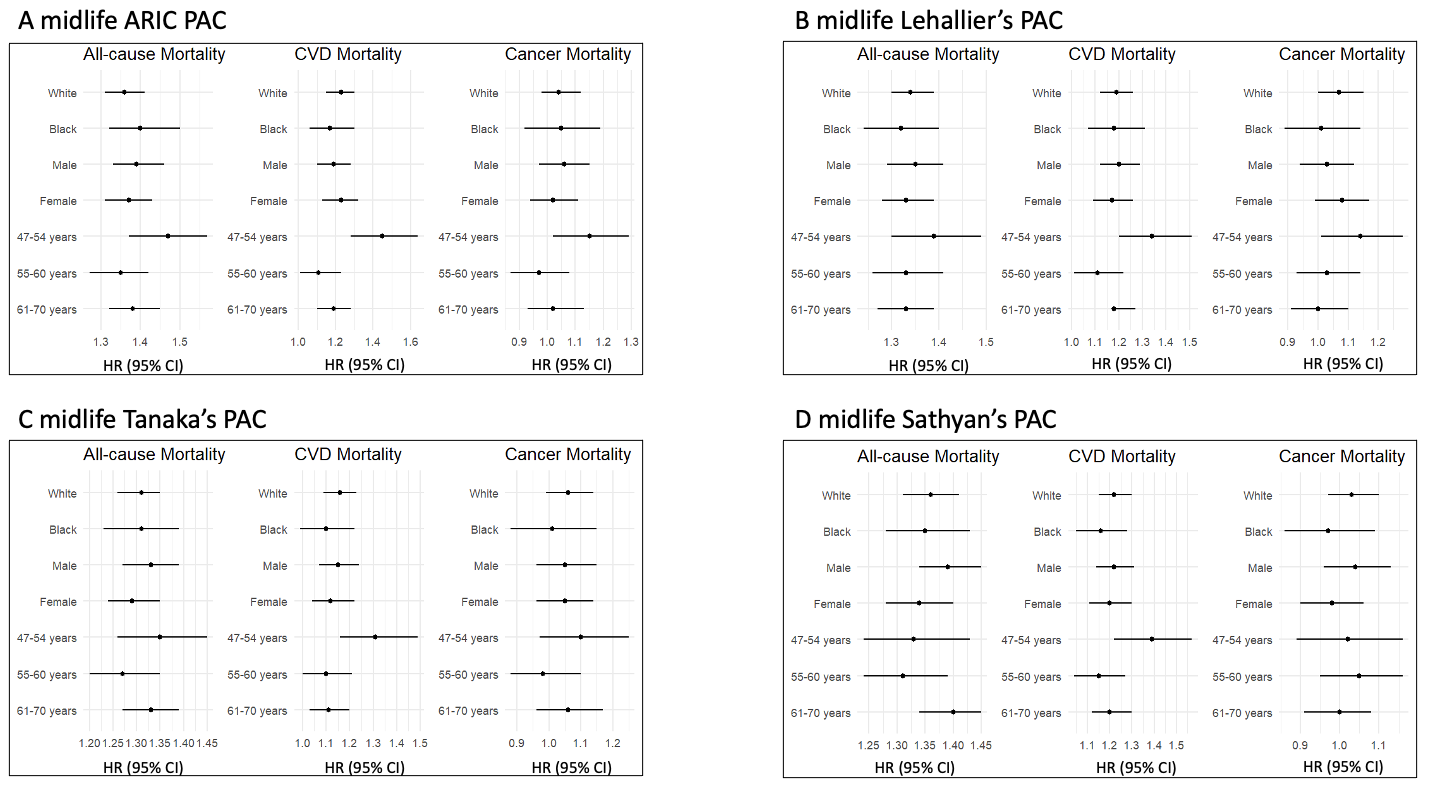

Supplement: S4 Fig — The plot shows the hazard ratio (HR) and 95% confidence interval (CI) for mortality with per one standard deviation (SD) increase in age acceleration for midlife ARIC PAC (A), midlife Lehallier’s PAC (B), midlife Tanaka’s PAC (C), and midlife Sathyan’s PAC (D). (TIF) [file pmed.1004464.s006.tif]

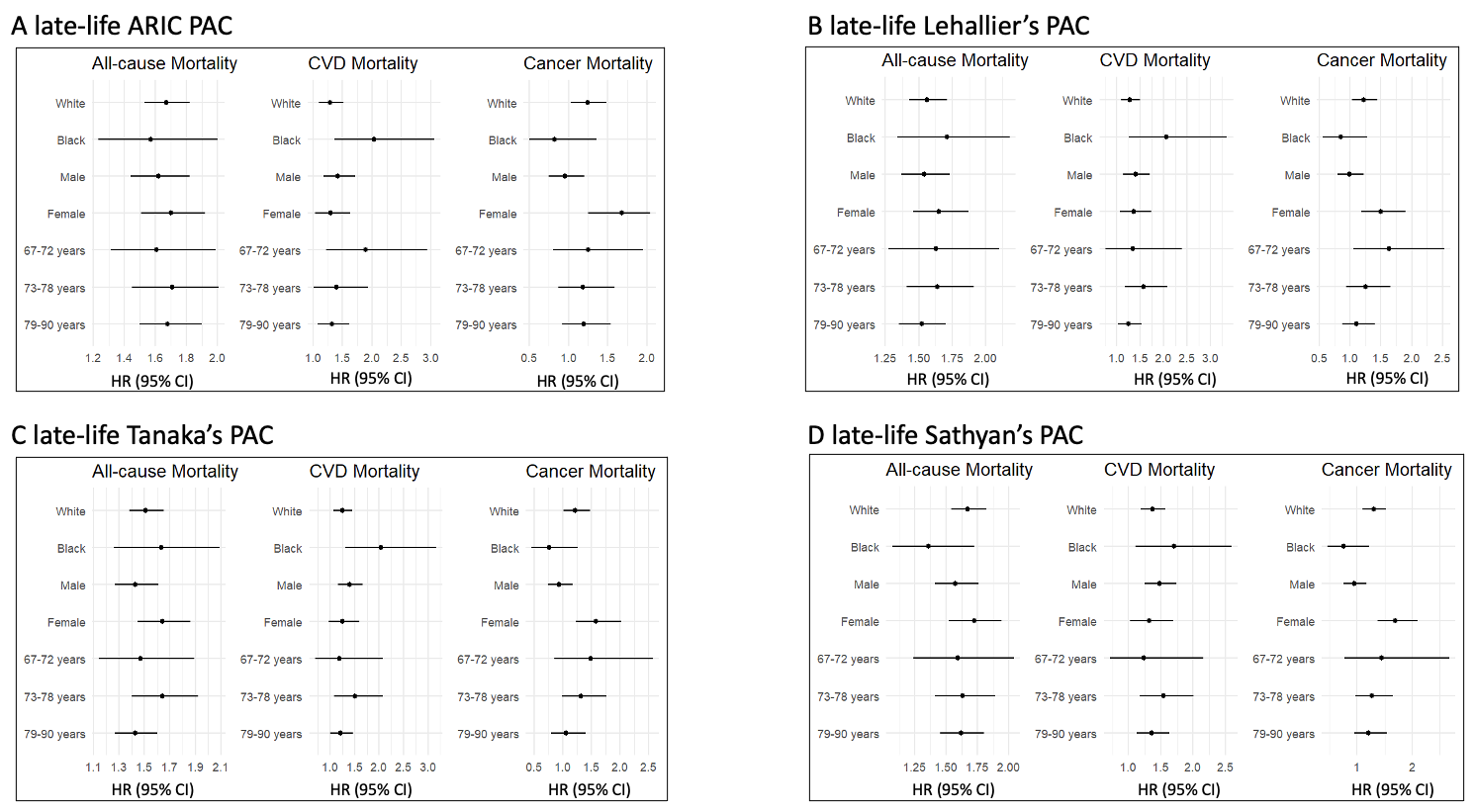

Supplement: S5 Fig — The plot shows the hazard ratio (HR) and 95% confidence interval (CI) for mortality with per one standard deviation (SD) increase in age acceleration for late-life ARIC PAC (A), late-life Lehallier’s PAC (B), late-life Tanaka’s PAC (C), and late-life Sathyan’s PAC (D). (TIF) [file pmed.1004464.s007.tif]
