# Supplementary material for: Development, characterization, and replication of proteomic aging clocks: Analysis of 2 population-based cohorts
Source: PLoS Med. 2024 Sep 24;21(9):e1004464. doi: 10.1371/journal.pmed.1004464 (PMC11460707; doi:10.1371/journal.pmed.1004464)
Supplement: S1 Table — (DOCX) [file pmed.1004464.s008.docx]

**S1 Table. Description of Lehallier’s, Tanaks’s, and Sathyan’s proteomic aging clocks (PACs)**

| PAC | Study population | Chronological age | SomaScan assay | Number of aptamers reported in PACs | Correlation between PAC and Chronological age reported in published papers | Number of aptamers available in ARIC among the aptamers reported in published PACs |
| --- | --- | --- | --- | --- | --- | --- |
| Lehallier’s PAC | 3,301 healthy individuals from the INTERVAL cohort (49% female; Individuals from Europe) | 18-76 years | SomaScan v.3 assay (measured 2,978 proteins) | 491 | 0.96 | 415  (85% of all 491 aptamers) |
|  |  |  |  |  |  |  |
| Tanaka’s PAC | 240 healthy individuals from the BLSA and GESTALT studies (50% female; White and Black individuals, as well as individuals from other race groups) | 22-93 years | SomaScan v.2 assay (measured 1,301 proteins) | 76 | 0.94 | 68  (89% of all 76 aptamers) |
|  |  |  |  |  |  |  |
| Sathyan’s PAC | 1,025 individuals from the LonGenity study (55.7% female; Individuals from Europe) | 65-95 years | SomaScan v.4 assay (measured 4,265 proteins) | 162 | 0.79 | 162  (100% of all 162 aptamers) |
