# Supplementary material for: Development, characterization, and replication of proteomic aging clocks: Analysis of 2 population-based cohorts
Source: PLoS Med. 2024 Sep 24;21(9):e1004464. doi: 10.1371/journal.pmed.1004464 (PMC11460707; doi:10.1371/journal.pmed.1004464)
Supplement: S2 Table — (DOCX) [file pmed.1004464.s009.docx]

**S2 Table. Description of the de novo ARIC proteomic aging clocks (PACs) constructed in midlife healthy participants; ARIC**

| PAC | Penalized regression | Transformation of proteins | Included square and cubic terms of each aptamer | Number of aptamers included in PAC |
| --- | --- | --- | --- | --- |
| midlife ARIC PAC | Elastic net regression (alpha=0.5) | Log2-transformed | No | 788 |
|  |  |  |  |  |
| midlife ARIC PAC LASSO | Lasso regression | Log2-transformed | No | 781 |
|  |  |  |  |  |
| midlife ARIC PAC winsorized | Elastic net regression (alpha=0.5) | Log2-transformed and then winsorized at 4 standard deviations (SD) | No | 631 |
|  |  |  |  |  |
| midlife ARIC PAC INT | Elastic net regression (alpha=0.5) | Log2-transformed and then applied inverse normal transformation (INT) | No | 694 |
|  |  |  |  |  |
| midlife ARIC PAC nonlinear | Elastic net regression (alpha=0.5) | Log2-transformed | Yes | 1071 (including the number of aptamers, square terms of aptamers, and cubic terms of aptamers) |
