# Supplementary material for: Development, characterization, and replication of proteomic aging clocks: Analysis of 2 population-based cohorts
Source: PLoS Med. 2024 Sep 24;21(9):e1004464. doi: 10.1371/journal.pmed.1004464 (PMC11460707; doi:10.1371/journal.pmed.1004464)
Supplement: S3 Table — (DOCX) [file pmed.1004464.s010.docx]

**S3 Table. Pearson correlation coefficients^a^ between the de novo ARIC proteomic aging clocks (PACs) constructed in midlife healthy participants and published PACs among the Visit 2 test set of healthy participants**

|  | midlife ARIC PAC | midlife ARIC PAC LASSO | midlife ARIC PAC winsorized | midlife ARIC PAC INT | midlife ARIC PAC nonlinear | midlife Lehallier’s PAC | midlife Tanaka’s PAC | midlife Sathyan’s PAC |
| --- | --- | --- | --- | --- | --- | --- | --- | --- |
| midlife ARIC PAC | 1.00 |  |  |  |  |  |  |  |
| midlife ARIC PAC LASSO | 1.00 | 1.00 |  |  |  |  |  |  |
| midlife ARIC PAC winsorized | 0.99 | 0.99 | 1.00 |  |  |  |  |  |
| midlife ARIC PAC INT | 0.97 | 0.97 | 0.98 | 1.00 |  |  |  |  |
| midlife ARIC PAC nonlinear | 0.99 | 0.99 | 0.99 | 0.98 | 1.00 |  |  |  |
| midlife Lehallier’s PAC | 0.89 | 0.89 | 0.90 | 0.90 | 0.89 | 1.00 |  |  |
| midlife Tanaka’s PAC | 0.77 | 0.77 | 0.78 | 0.77 | 0.77 | 0.79 | 1.00 |  |
| midlife Sathyan’s PAC | 0.71 | 0.71 | 0.72 | 0.71 | 0.71 | 0.68 | 0.67 | 1.00 |
| ^a^All p-values for Pearson correlation coefficients were <0.001. | | | | | | | | |
