# Supplementary material for: Development, characterization, and replication of proteomic aging clocks: Analysis of 2 population-based cohorts
Source: PLoS Med. 2024 Sep 24;21(9):e1004464. doi: 10.1371/journal.pmed.1004464 (PMC11460707; doi:10.1371/journal.pmed.1004464)
Supplement: S4 Table — (DOCX) [file pmed.1004464.s011.docx]

**S4 Table. Including/excluding participants with controlled hypertension^a^ for late-life healthy participants at Visit 5 to construct proteomic aging clocks (PACs) using elastic net regression**

|  | Participants with uncontrolled hypertension are excluded for healthy participants (the late-life ARIC PAC) | Participants with (controlled and uncontrolled) hypertension are excluded for healthy participants |
| --- | --- | --- |
| No. of healthy participants | 945 | 483 |
| Training set size | 630 | 322 |
| Test set size | 315 | 161 |
| Pearson correlation^b^ in the training set | 0.84 (p<0.001) | 0.86 (p<0.001) |
| Pearson correlation^b^ in the test set | 0.71 (p<0.001) | 0.64 (p<0.001) |
| Median absolute error (MAE)^b^ in the training set | 1.47 | 1.89 |
| MAE^b^ in the test set | 2.36 | 2.47 |
| Standard deviation (SD) for age acceleration | 2.61 | 2.47 |
| HR (95% CI)^c^ for all-cause mortality until 2019 per one SD increase in age acceleration^d^ | 1.65 (1.52, 1.79), p<0.001 | 1.66 (1.54, 1.78), p<0.001 |
| ^a^Hypertension was defined as diastolic blood pressure ≥90 mmHg, systolic blood pressure ≥140 mmHg, or taking medication for high blood pressure. Hypertension is controlled if the measured diastolic blood pressure is below 90 and the measured systolic blood pressure is below 140 while the participant is on medication. | | |
| ^b^Pearson correlation between PAC and chronological age. | | |
| ^c^The Model was adjusted for chronological age, sex, joint terms for race and study center (Black participants from Mississippi; Black participants from any other centers; White participants from Maryland; White participants from North Carolina; and White participants from Minnesota), education, body mass index (BMI), smoking status, pack-years of smoking, alcohol intake, physical activity, diabetes, hypertension, cardiovascular disease (CVD), and estimated glomerular filtration rate (eGFR) at Visit 5. | | |
| ^d^Age acceleration for each PAC was calculated as residuals after regressing PAC on chronological age. | | |
