# Supplementary material for: Development, characterization, and replication of proteomic aging clocks: Analysis of 2 population-based cohorts
Source: PLoS Med. 2024 Sep 24;21(9):e1004464. doi: 10.1371/journal.pmed.1004464 (PMC11460707; doi:10.1371/journal.pmed.1004464)
Supplement: S5 Table — (DOCX) [file pmed.1004464.s012.docx]

**S5 Table. R squared after regressing age acceleration for the midlife and late-life ARIC proteomic aging clocks (PACs) on covariates at the corresponding visits**

| **Regressing age acceleration for the midlife ARIC PAC on Visit 2 covariates** | |
| --- | --- |
| Participants characteristics at Visit 2 | R squared |
| Chronological age | 0.0000 |
| Chronological age +sex | 0.0000 |
| Chronological age + joint terms of race and center | 0.0039 |
| Chronological age + education | 0.0074 |
| Chronological age + body mass index (BMI) | 0.0011 |
| Chronological age + smoking, pack-years of smoking | 0.0032 |
| Chronological age + alcohol intake | 0.0051 |
| Chronological age + physical activity at Visit 1 | 0.0064 |
| Chronological age + aspirin use | 0.0006 |
| Chronological age + hormone replacement therapy | 0.0017 |
| Chronological age + diabetes | 0.0264 |
| Chronological age + estimated glomerular filtration rate (eGFR) | 0.0778 |
| Chronological age + hypertension | 0.0124 |
| Chronological age + cardiovascular disease (CVD) | 0.0166 |
| **Regressing age acceleration for the late-life ARIC PAC on Visit 5 covariates** | |
| Participants characteristics at Visit 5 | R squared |
| Chronological age | 0.0000 |
| Chronological age +sex | 0.0023 |
| Chronological age + joint terms of race and center | 0.0037 |
| Chronological age + education | 0.0020 |
| Chronological age + BMI | 0.0016 |
| Chronological age + smoking, pack-years of smoking | 0.0089 |
| Chronological age + alcohol intake | 0.0035 |
| Chronological age + physical activity | 0.0292 |
| Chronological age + aspirin use | 0.0013 |
| Chronological age + diabetes | 0.0001 |
| Chronological age + eGFR | 0.2033 |
| Chronological age + hypertension | 0.0046 |
| Chronological age + CVD | 0.0316 |
