# Supplementary material for: Development, characterization, and replication of proteomic aging clocks: Analysis of 2 population-based cohorts
Source: PLoS Med. 2024 Sep 24;21(9):e1004464. doi: 10.1371/journal.pmed.1004464 (PMC11460707; doi:10.1371/journal.pmed.1004464)
Supplement: S6 Table — (DOCX) [file pmed.1004464.s013.docx]

S6 Table. Pearson correlation between Tanaka’s and Sathyan’s proteomic aging clocks (PACs) and chronological age and median absolute error (MAE); ARIC

| ***Midlife -- Visit 2 (N = 2,993 in training set N = 1,496 in test set)*** | | |
| --- | --- | --- |
|  | midlife Tanaka’s PAC | midlife Sathyan’s PAC^b^ |
| Lambda value | 0.19 | - |
| Correlation in the training set^a^ | 0.68 (p<0.001) | - |
| Correlation in the test set^b^ | 0.66 (p<0.001) | 0.58 (p<0.001) |
| MAE in the training set^b^ | 2.74 | - |
| MAE in the test set^b^ | 2.78 | 10.00 |
| ***Late life -- Visit 5 (N = 630 in training set N = 315 in test set)*** | | |
|  | late-life Tanakas’ PAC | late-life Sathyan’s PAC^b^ |
| Lambda value | 1.32 | -- |
| Correlation in the training set^a^ | 0.69 (p<0.001) | -- |
| Correlation in the test set^a^ | 0.59 (p<0.001) | 0.70 (p<0.001) |
| MAE in the training set^b^ | 2.09 |  |
| MAE in the test set^a^ | 2.48 | 2.34 |
| ^a^Among healthy participants at Visit 2 and Visit 5, we randomly selected two-thirds of healthy participants at each visit and used them as the training set at the corresponding visits; the remaining one-third of healthy participants at each visit was used as the test set at the corresponding visits. | | |
| ^b^Sathyan’s PAC was computed using the published weights. We only calculated the correlation between Sathyan’s PAC and chronological age and MAE in the test set. | | |
