# Supplementary material for: Development, characterization, and replication of proteomic aging clocks: Analysis of 2 population-based cohorts
Source: PLoS Med. 2024 Sep 24;21(9):e1004464. doi: 10.1371/journal.pmed.1004464 (PMC11460707; doi:10.1371/journal.pmed.1004464)
Supplement: S7 Table — (DOCX) [file pmed.1004464.s014.docx]

| **S7 Table. Visit 2 participants’ characteristics across quartiles of age acceleration for midlife Tanaka’s and Sathyan’s PACs; ARIC** | | | | | | | | | | |
| --- | --- | --- | --- | --- | --- | --- | --- | --- | --- | --- |
|  | **midlife Tanaka's PAC** | | | | | **midlife Sathyan's PAC** | | | | |
|  | Q1  (N=2,192) | Q2  (N=2,192) | Q3  (N=2,192) | Q4  (N=2,192) | P-value^c^ | Q1  (N=2,192) | Q2  (N=2,192) | Q3  (N=2,192) | Q4  (N=2,192) | P-value^c^ |
| Age acceleration, years | -16.7 to -2.0 | -1.9 to -0.2 | -0.1 to +1.7 | +1.8 to +28.4 |  | -8.8 to -1.7 | -1.7 to -0.1 | -0.1 to +1.6 | +1.6 to +24.0 |  |
| Mean age acceleration, years | -3.68 | -1.06 | 0.74 | 3.99 |  | -3.15 | -0.94 | 0.66 | 3.44 |  |
| Chronological age, years (SD) | 58.3 (5.8) | 58.1 (5.7) | 57.6 (5.6) | 58.2 (5.6) | <0.001 | 58.2 (5.5) | 57.9 (5.7) | 58.1 (5.8) | 58.2 (5.9) | 0.295 |
| Female, % | 56.4 | 53.7 | 53.7 | 54.6 | 0.226 | 50.5 | 51.6 | 55.6 | 60.8 | <0.001 |
| White, % | 64.8 | 74.8 | 76.0 | 76.1 | <0.001 | 63.3 | 72.5 | 78.6 | 77.4 | <0.001 |
| Education, % |  |  |  |  |  |  |  |  |  |  |
| <High school | 23.6 | 23.2 | 22.7 | 27.0 | <0.001 | 24.4 | 22.8 | 23.2 | 25.0 | 0.044 |
| High school/vocational | 39.2 | 42.0 | 42.7 | 41.8 |  | 38.6 | 42.8 | 42.5 | 42.3 |  |
| College | 37.2 | 34.7 | 34.5 | 31.2 |  | 36.9 | 34.4 | 34.3 | 32.7 |  |
| BMI, kg/m^2^ (SD) | 29.0 (5.7) | 28.5 (5.3) | 28.3 (5.4) | 27.8 (5.6) | <0.001 | 29.2 (5.1) | 28.5 (5.2) | 28.3 (5.5) | 27.5 (6.2) | <0.001 |
| Smoking status, % |  |  |  |  |  |  |  |  |  |  |
| Current smoker | 18.3 | 21.1 | 24.8 | 27.8 | <0.001 | 19.6 | 23.0 | 22.6 | 26.5 | 0.001 |
| Former smoker | 36.5 | 40.2 | 38.5 | 38.5 |  | 39.6 | 39.8 | 39.0 | 35.9 |  |
| Never smoker | 45.2 | 38.6 | 36.7 | 33.6 |  | 40.8 | 37.2 | 38.4 | 37.6 |  |
| Pack-years of smoking among ever smokers, pack-years (SD) | 27.8 (22.9) | 28.7 (22.6) | 31.1 (22.5) | 33.2 (23.9) | <0.001 | 28.0 (22.2) | 29.3 (23.3) | 30.7 (22.7) | 33.3 (24.0) | <0.001 |
| Alcohol intake, % |  |  |  |  |  |  |  |  |  |  |
| Current drinker | 54.2 | 56.4 | 55.4 | 52.9 | 0.165 | 57.3 | 56.4 | 56.3 | 49.1 | <0.001 |
| Former drinker | 22.1 | 21.7 | 21.6 | 24.3 |  | 21.6 | 22.1 | 21.1 | 24.6 |  |
| Never drinker | 23.7 | 21.9 | 22.9 | 22.7 |  | 21.0 | 21.5 | 22.6 | 26.2 |  |
| Physical activity, score^a^ (SD) | 2.42 (0.8) | 2.44 (0.8) | 2.43 (0.8) | 2.39 (0.8) | 0.196 | 2.44 (0.8) | 2.45 (0.8) | 2.43 (0.8) | 2.36 (0.8) | <0.01 |
| Aspirin use in the preceding two weeks, % | 47.6 | 52.2 | 52.1 | 57.7 | <0.001 | 49.6 | 49.6 | 54.4 | 55.1 | <0.001 |
| Ever user for hormone replacement therapy (females only), % | 47.8 | 45.6 | 41.8 | 39.0 | <0.001 | 52.6 | 47.7 | 40.2 | 34.2 | <0.001 |
| Diabetes^b^, % | 22.4 | 19.8 | 18.6 | 20.7 | 0.018 | 25.2 | 18.1 | 18.3 | 19.5 | <0.001 |
| Hypertension^b^, % | 47.9 | 47.6 | 47.4 | 51.2 | 0.037 | 49.7 | 47.2 | 46.9 | 49.3 | 0.173 |
| CVD^b^, % | 11.5 | 13.3 | 15.2 | 23.7 | <0.001 | 12.6 | 13.4 | 14.9 | 20.6 | <0.001 |
| eGFR, mL/min/1.73 m^2^ (SD) | 98.7 (11.6) | 97.4 (12.5) | 96.3 (12.9) | 90.2 (19.7) | <0.001 | 98.1(11.6) | 97.4(12.4) | 95.6(13.8) | 91.4(19.5) | <0.001 |
| Abbreviations: PAC – proteomic aging clock; SD – standard deviation; BMI - body mass index; CVD – cardiovascular disease; eGFR – estimated glomerular filtration rate. | | | | | | | | | | |
| ^a^Physical activity at Visit 1 was assessed using a leisure-time sprots index that ranged from 1 to 5. We assumed that physical activity scores were the same at Visit 1 and Visit 2. We reported physical activity scores with two decimal places to illustrate the trend more effectively. | | | | | | | | | | |
| ^b^All diseases are prevalent diseases. | | | | | | | | | | |
| ^c^P-values were calculated using chi-square tests for categorical variables and using ANOVA tests for continuous variables. | | | | | | | | | | |
