# Supplementary material for: Development, characterization, and replication of proteomic aging clocks: Analysis of 2 population-based cohorts
Source: PLoS Med. 2024 Sep 24;21(9):e1004464. doi: 10.1371/journal.pmed.1004464 (PMC11460707; doi:10.1371/journal.pmed.1004464)
Supplement: S8 Table — (DOCX) [file pmed.1004464.s015.docx]

**S8 Table. The association between age acceleration for midlife Tanaka’s and Sathyan’s PACs and mortality; ARIC (1990-2019)**

|  | No. of participants | No. of deaths | Total person-years | **midlife Tanaka’s PAC** | p-value | **midlife Sathyan’s PAC** | p-value |
| --- | --- | --- | --- | --- | --- | --- | --- |
|  |  |  |  | HR (95% CI)^a^ per 1 SD  of age acceleration  (SD=3.15 years) |  | HR (95% CI)^a^ per 1 SD  of age acceleration  (SD=2.70 years) |  |
| All-cause mortality | 8,768 | 5,294 | 182,630 | 1.31 (1.27, 1.35) | <0.001 | 1.34 (1.30, 1.38) | <0.001 |
| CVD mortality (Fine and Gray model) | 8,768 | 1,734 | 182,630 | 1.14 (1.08, 1.20) | <0.001 | 1.19 (1.13, 1.26) | <0.001 |
| Cancer mortality (Fine and Gray model) | 8,768 | 1,516 | 182,630 | 1.05 (0.98, 1.11) | 0.211 | 1.01 (0.95, 1.07) | 0.733 |
| LRD mortality (Find and Gray model) | 8,768 | 522 | 182,630 | 1.25 (1.12, 1.39) | <0.001 | 1.30 (1.18, 1.44) | <0.001 |
| Abbreviations: PAC – proteomic aging clock; BMI – body mass index; CVD – cardiovascular disease; LRD – lower respiratory disease; eGFR – estimated glomerular filtration rate; SD – standard deviation; HR – Hazard ratio; CI – confidence interval. | | | | | | | |
| ^a^ The model was adjusted for chronological age, sex, joint terms for race and study center (Black participants from Mississippi; Black participants from any other centers; White participants from Maryland; White participants from North Carolina; and White participants from Minnesota), education, BMI, smoking status, pack-years of smoking, alcohol intake, physical activity (at Visit 1), hormone replacement therapy, diabetes, hypertension, CVD, and eGFR at Visit 2. | | | | | | | |
