# Supplementary material for: Development, characterization, and replication of proteomic aging clocks: Analysis of 2 population-based cohorts
Source: PLoS Med. 2024 Sep 24;21(9):e1004464. doi: 10.1371/journal.pmed.1004464 (PMC11460707; doi:10.1371/journal.pmed.1004464)
Supplement: S9 Table — (DOCX) [file pmed.1004464.s016.docx]

**S9 Table. Pearson correlation coefficients^a^ between the late-life ARIC PAC and published PACs in the Visit 5 test set of healthy participants**

|  | late-life ARIC PAC | late-life Lehallier’s PAC | | late-life Tanaka’s PAC | late-life Sathyan’s PAC |
| --- | --- | --- | --- | --- | --- |
| late-life ARIC PAC | 1.00 |  |  | |  |
| late-life Lehallier’s PAC | 0.84 | 1.00 |  | |  |
| late-life Tanaka’s PAC | 0.79 | 0.78 | 1.00 | |  |
| late-life Sathyan’s PAC | 0.84 | 0.78 | 0.77 | | 1.00 |
| ^a^ All p-values for Pearson correlation coefficients were <0.001. | | | | | |
