# Supplementary material for: Development, characterization, and replication of proteomic aging clocks: Analysis of 2 population-based cohorts
Source: PLoS Med. 2024 Sep 24;21(9):e1004464. doi: 10.1371/journal.pmed.1004464 (PMC11460707; doi:10.1371/journal.pmed.1004464)
Supplement: S10 Table — (DOCX) [file pmed.1004464.s017.docx]

| **S10 Table. Visit 5 participants’ characteristics across quartiles of age acceleration for late-life Tanaka’s and Sathyan’s PACs; ARIC** | | | | | | | | | | |
| --- | --- | --- | --- | --- | --- | --- | --- | --- | --- | --- |
|  | **late-life Tanaka's PAC** | | | | | **late-life Sathyan's PAC** | | | | |
|  | Q1  (N = 1,138) | Q2  (N = 1,138) | Q3  (N = 1,138) | Q4  (N = 1,138) | P-value^c^ | Q1  (N = 1,138) | Q2  (N = 1,138) | Q3  (N = 1,139) | Q4  (N = 1,138) | P-value^c^ |
| Age acceleration (min to max, years | -9.3 to -2.0 | -2.0 to -0.3 | -0.3 to 1.6 | 1.6 to 17.0 |  | -10.4 to -2.1 | -2.1 to -0.1 | -0.1 to +2.1 | 2.1 to +21.3 |  |
| Mean age acceleration, years | -3.43 | -1.05 | 0.55 | 3.82 |  | -3.85 | -1.22 | 0.78 | 4.30 |  |
| Chronological age, years (SD) | 76.8 (5.0) | 76.0 (5.0) | 76.3 (5.1) | 76.8 (5.3) | <0.001 | 77.0 (5.2) | 76.1 (5.0) | 76.0 (5.2) | 77.0 (5.5) | <0.001 |
| Female, % | 62.0 | 59.8 | 54.4 | 48.2 | <0.001 | 49.0 | 54.2 | 60.9 | 61.2 | <0.001 |
| White, % | 76.2 | 80.2 | 82.3 | 82.4 | <0.001 | 69.6 | 80.7 | 84.6 | 86.2 | <0.001 |
| Education, % |  |  |  |  |  |  |  |  |  |  |
| <High school | 13.1 | 12.4 | 15.1 | 17.0 | 0.020 | 15.5 | 11.9 | 13.6 | 16.8 | <0.001 |
| High school/vocational | 41.7 | 43.2 | 42.5 | 43.1 |  | 37.9 | 43.9 | 44.8 | 43.8 |  |
| College | 45.2 | 44.4 | 42.4 | 39.9 |  | 46.6 | 44.2 | 41.6 | 39.4 |  |
| BMI, kg/m^2^ (SD) | 29.3 (5.5) | 29.0 (5.6) | 28.7 (5.8) | 28.2 (5.7) | <0.001 | 29.3 (5.2) | 28.7 (5.4) | 28.8 (5.8) | 28.2 (6.2) | <0.001 |
| Smoking status, % |  |  |  |  |  |  |  |  |  |  |
| Current smoker | 4.6 | 5.5 | 7.0 | 8.8 | <0.001 | 4.8 | 5.3 | 7.3 | 8.5 | 0.002 |
| Former smoker | 51.5 | 52.9 | 53.4 | 54.7 |  | 56.6 | 53.4 | 50.0 | 52.4 |  |
| Never smoker | 43.9 | 41.6 | 39.6 | 36.5 |  | 38.6 | 41.3 | 42.7 | 39.1 |  |
| Pack-years of smoking among ever smokers, pack-years (SD) | 10.2 (16.4) | 11.4 (18.8) | 13.3 (20.3) | 16.1 (23.5) | <0.001 | 12.5 (18.6) | 10.8 (18.2) | 12.1 (20.4) | 15.5 (22.2) | <0.001 |
| Alcohol intake, % |  |  |  |  |  |  |  |  |  |  |
| Current drinker | 49.6 | 49.4 | 50.0 | 50.3 | 0.578 | 51.7 | 50.9 | 49.4 | 47.1 | 0.073 |
| Former drinker | 31.2 | 28.6 | 28.7 | 30.1 |  | 30.5 | 28.6 | 28.2 | 31.3 |  |
| Never drinker | 19.2 | 22.0 | 21.3 | 19.6 |  | 17.8 | 20.5 | 22.4 | 21.6 |  |
| Physical activity ^a^, scores (SD) | 2.67 (0.8) | 2.62 (0.8) | 2.57 (0.8) | 2.42 (0.7) | <0.001 | 2.69 (0.8) | 2.68 (0.8) | 2.54 (0.8) | 2.36 (0.8) | <0.001 |
| Aspirin use in the preceding two weeks, % | 65.7 | 70.6 | 71.6 | 74.7 | <0.001 | 69.6 | 70.0 | 69.8 | 73.2 | 0.183 |
| Diabetes^b^, % | 36.1 | 36.7 | 37.3 | 40.0 | 0.248 | 40.9 | 35.7 | 35.8 | 37.6 | 0.036 |
| Hypertension^b^, % | 73.0 | 74.5 | 78.0 | 82.5 | <0.001 | 74.8 | 77.5 | 75.4 | 80.3 | 0.009 |
| CVD^b^, % | 17.9 | 22.6 | 30.2 | 42.0 | <0.001 | 21.6 | 25.2 | 28.4 | 37.7 | <0.001 |
| eGFR, mL/min/1.73 m^2^ (SD) | 77.4 (14.1) | 74.2 (15.3) | 69.6 (16.7) | 60.7 (20.1) | <0.001 | 76.3 (15.0) | 73.8 (14.9) | 70.5 (17.0) | 61.4 (20.3) | <0.001 |
| Abbreviations: PAC – proteomic aging clock; SD – standard deviation; BMI - body mass index; CVD – cardiovascular disease; eGFR – estimated glomerular filtration rate. | | | | | | | | | | |
| ^a^Physical activity was assessed using a leisure-time sprots index score that ranged from 1 to 5. We reported physical activity scores with two decimal places to illustrate the trend more effectively. | | | | | | | | | | |
| ^b^All diseases are prevalent diseases. | | | | | | | | | | |
| ^c^P-values were calculated using chi-square tests for categorical variables and using ANOVA tests for continuous variables. | | | | | | | | | | |
