# Supplementary material for: Development, characterization, and replication of proteomic aging clocks: Analysis of 2 population-based cohorts
Source: PLoS Med. 2024 Sep 24;21(9):e1004464. doi: 10.1371/journal.pmed.1004464 (PMC11460707; doi:10.1371/journal.pmed.1004464)
Supplement: S11 Table — (DOCX) [file pmed.1004464.s018.docx]

S11 Table. The associations of age acceleration for late-life Tanaka’s and Sathyan’s PACs with mortality; ARIC (2011-2019)

|  | No. of participants | No. of deaths | Total person-years | **late-life Tanakas’ PAC**  HR (95%CI) ^a^ per 1 SD  of age acceleration  (SD = 2.90 years) | p-value | **late-life Sathyan’s PAC**  HR (95%CI) ^a^ per 1 SD  of age acceleration  (SD = 3.28 years) | p-value |
| --- | --- | --- | --- | --- | --- | --- | --- |
| All-cause mortality | 4,553 | 1,123 | 29,356 | 1.53 (1.40, 1.67) | <0.001 | 1.65 (1.52, 1.79) | <0.001 |
| CVD mortality (Fine and Gray model) | 4,553 | 348 | 29,356 | 1.33 (1.15, 1.54) | <0.001 | 1.42 (1.23, 1.63) | <0.001 |
| Cancer mortality (Fine and Gray model) | 4,553 | 278 | 29,356 | 1.19 (0.99, 1.43) | 0.066 | 1.25 (1.06, 1.47) | 0.031 |
| LRD mortality (Fine and Gray model) | 4,553 | 128 | 29,356 | 1.42 (1.13, 1.80) | <0.001 | 1.49 (1.19, 1.87) | <0.001 |
| Abbreviations: PAC – proteomic aging clock; SD – standard deviation; BMI – body mass index; CVD – cardiovascular disease; LRD – lower respiratory disease; eGFR – estimated glomerular filtration rate. HR – Hazard ratio; CI – confidence interval. | | | | | | | |
| ^a^ The model was adjusted for chronological age, sex, joint terms for race and study center (Black participants from Mississippi; Black participants from any other centers; White participants from Maryland; White participants from North Carolina; and White participants from Minnesota), education, BMI, smoking status, pack-years of smoking, alcohol intake, physical activity, diabetes, hypertension, CVD, and eGFR at Visit 5. | | | | | | | |
