# Supplementary material for: Development, characterization, and replication of proteomic aging clocks: Analysis of 2 population-based cohorts
Source: PLoS Med. 2024 Sep 24;21(9):e1004464. doi: 10.1371/journal.pmed.1004464 (PMC11460707; doi:10.1371/journal.pmed.1004464)
Supplement: S12 Table — (DOCX) [file pmed.1004464.s019.docx]

S12 Table. The associations of age acceleration for Sathyan’s PAC with mortality in all ARIC participants

|  | ***In all V2 participants*** | | | | | |
| --- | --- | --- | --- | --- | --- | --- |
|  | | No. of participants | No. of deaths | Total person-years | midlife Sathyan’s PAC  HR (95%CI) ^a^ per 1 SD  (SD = 2.60 years) | p-value |
| All-cause mortality | | 11,761 | 6,418 | 255,858 | 1.33 (1.29, 1.36) | <0.001 |
| CVD mortality (Fine and Gray model) | | 11,761 | 1,994 | 255,858 | 1.20 (1.14, 1.26) | <0.001 |
| Cancer mortality (Fine and Gray model) | | 11,761 | 1,888 | 255,858 | 1.01 (0.96, 1.06) | 0.708 |
| LRD mortality (Fine and Gray model) | | 11,761 | 599 | 255,858 | 1.29 (1.18, 1.42) | <0.001 |
|  | ***In all V5 participants*** | | | | | |
|  | | No. of participants | No. of deaths | Total person-years | late-life Sathyan’s PAC  HR (95%CI) ^a^ per 1 SD  (SD = 3.23 years) | p-value |
| All-cause mortality | | 5,183 | 1,185 | 33,875 | 1.64 (1.52, 1.77) | <0.001 |
| CVD mortality (Fine and Gray model) | | 5,183 | 362 | 33,875 | 1.44 (1.25, 1.65) | <0.001 |
| Cancer mortality (Fine and Gray model) | | 5,183 | 294 | 33,875 | 1.22 (1.04, 1.43) | 0.014 |
| LRD mortality (Fine and Gray model) | | 5,183 | 131 | 33,875 | 1.57 (1.26, 1.95) | <0.001 |
| Abbreviations: PAC – proteomic aging clock; SD – standard deviation; BMI – body mass index; CVD – cardiovascular disease; LRD – lower respiratory disease; eGFR – estimated glomerular filtration rate. HR – Hazard ratio; CI – confidence interval. | | | | | | |
| ^a^ The Model was adjusted for chronological age, sex, joint terms for race and study center (Black participants from Mississippi; Black participants from any other centers; White participants from Maryland; White participants from North Carolina; and White participants from Minnesota) education, BMI, smoking status, pack-years of smoking, alcohol intake, physical activity, hormone replacement therapy (at Visit 2 only), diabetes, hypertension, CVD, and eGFR at corresponding visits. | | | | | | |
