# Supplementary material for: Development, characterization, and replication of proteomic aging clocks: Analysis of 2 population-based cohorts
Source: PLoS Med. 2024 Sep 24;21(9):e1004464. doi: 10.1371/journal.pmed.1004464 (PMC11460707; doi:10.1371/journal.pmed.1004464)
Supplement: S13 Table — (DOCX) [file pmed.1004464.s020.docx]

| **S13 Table. Common proteins included in published PACs and either the midlife or the late-life ARIC PACs** | |
| --- | --- |
| Protein | Functions of proteins^a^ |
| Pleiotrophin (PTN) | **Regulates processes like cell proliferation, cell survival, cell growth, cell differentiation and cell migration** |
|  |  |
| A disintegrin and metalloproteinase with thrombospondin motifs 5 (ADAMTS-5) | Play an important role in connective tissue organization, development, inflammation, arthritis, and cell migration |
|  |  |
| Macrophage metalloelastase (MMP12) | Involved in the breakdown of extracellular matrix in normal physiological processes |
|  |  |
| Cell adhesion molecule-related/down-regulated by oncogenes (CDON). | A member of immunoglobulin superfamily |
|  |  |
| Growth differentiation factor 15 (GDF15) | Regulates food intake, energy expenditure and body weight in response to metabolic and toxin-induced stresses |
|  |  |
| Immunoglobulin superfamily containing leucine-rich repeat protein 2 (ISLR2) | Required for axon extension during neural development |
|  |  |
| Kallikrein-7 (KLK7) | Could play a role in the activation of precursors to inflammatory cytokines. |
|  |  |
| Lactoperoxidase (LPO) | May contribute to airway host defense against infection. |
|  |  |
| R-spondin-4 (RSPO4) | Activator of the canonical Wnt signaling pathway |
|  |  |
| Growth differentiation factor 15 (GDF15) | Regulates food intake, energy expenditure and body weight in response to metabolic and toxin-induced stresses |
|  |  |
| Immunoglobulin superfamily containing leucine-rich repeat protein 2 (ISLR2) | Required for axon extension during neural development |
| ^a^Fuctions of proteins documented either in STRING (<https://string-db.org/>) or GeneCards (<https://www.genecards.org/>). | |
