# Supplementary material for: Development, characterization, and replication of proteomic aging clocks: Analysis of 2 population-based cohorts
Source: PLoS Med. 2024 Sep 24;21(9):e1004464. doi: 10.1371/journal.pmed.1004464 (PMC11460707; doi:10.1371/journal.pmed.1004464)
Supplement: S14 Table — (DOCX) [file pmed.1004464.s021.docx]

S14 Table. Top 20 proteins with the largest absolute weight in the midlife and late-life ARIC PACs

|  | | **midlife ARIC PAC** | | | |
| --- | --- | --- | --- | --- | --- |
| No. | Aptamer ID | | Proteins^a^ | Target | Weight |
| 1 | SeqId_8956_96 | | Scavenger receptor class F member 2 | SREC-II | 2.39462492 |
| 2 | SeqId_14136_234 | | Complement component C1q receptor | C1QR1 | -2.232162 |
| 3 | SeqId_16890_37 | | ADAMTS-like protein 1 | ATL1 | 1.9584385 |
| 4 | SeqId_15640_54 | | Transgelin | TAGL | 1.86624409 |
| 5 | SeqId_3045_72 | | Pleiotrophin | PTN | 1.79477603 |
| 6 | SeqId_3362_61 | | Chordin-like protein 1 | CRDK1 | 1.76344784 |
| 7 | SeqId_6392_7 | | WNT1-inducible-signaling pathway protein 2 | WISP-2 | 1.71303183 |
| 8 | SeqId_9793_145 | | Immunoglobulin superfamily DCC subclass member 4 | IGDC4 | -1.5215087 |
| 9 | SeqId_13114_50 | | Lumican | Lumican | 1.3807798 |
| 10 | SeqId_3331_8 | | RGM domain family member B | RGMB | -1.1463758 |
| 11 | SeqId_7179_69 | | Neurofascin | NFASC | 1.13848556 |
| 12 | SeqId_11196_31 | | Collagen alpha-3(VI) chain | Collagen alpha-3(VI) | -1.1215024 |
| 13 | SeqId_7551_33 | | Transforming growth factor beta activator LRRC32 | LRC32 | 1.11417136 |
| 14 | SeqId_8974_172 | | Collagen alpha-1(XV) chain | COFA1 | -1.0493308 |
| 15 | SeqId_3344_60 | | Antithrombin-III | Antithrombin III | -1.036693 |
| 16 | SeqId_12417_46 | | EKC/KEOPS complex subunit TPRKB | TPRKB | 1.02886089 |
| 17 | SeqId_8841_65 | | Cartilage intermediate layer protein 2 | CILP2 | -1.0021828 |
| 18 | SeqId_4541_49 | | Cell adhesion molecule-related/down-regulated by oncogenes | CDON | -0.9964351 |
| 19 | SeqId_9484_75 | | Desmoglein-2 | Desmoglein-2 | -0.994248 |
| 20 | SeqId_4374_45 | | Growth/differentiation factor 15 | MIC-1 | 0.97937678 |
| **late-life ARIC PAC** | | | | | |
| 1 | SeqId_2677_1 | | Protein flightless-1 homolog | ERBB1 | -2.522735 |
| 2 | SeqId_15640_54 | | Transgelin | TAGL | 2.17188056 |
| 3 | SeqId_6392_7 | | WNT1-inducible-signaling pathway protein 2 | WISP-2 | 2.03908149 |
| 4 | SeqId_3362_61 | | Chordin-like protein 1 | CRDL1 | 1.48881636 |
| 5 | SeqId_3045_72 | | Pleiotrophin | PTN | 1.39529172 |
| 6 | SeqId_8974_172 | | Collagen alpha-1(XV) chain | COFA1 | -1.032661 |
| 7 | SeqId_14136_234 | | Complement component C1q receptor | C1QR1 | -0.9259469 |
| 8 | SeqId_6081_52 | | Aldo-keto reductase family 1 member B10 | PCOC2 | -0.7204833 |
| 9 | SeqId_19332_1 | | MOB-like protein phocein | MOBL3 | 0.66952444 |
| 10 | SeqId_15434_5 | | Prepronociceptin | PNOC | 0.63560224 |
| 11 | SeqId_11388_75 | | WAP four-disulfide core domain protein 2 | HE4 | 0.60409029 |
| 12 | SeqId_8480_29 | | EGF-containing fibulin-like extracellular matrix protein 1 | FBLN3 | 0.59731828 |
| 13 | SeqId_8304_50 | | Tumor necrosis factor receptor superfamily member 11B | OPG | 0.56012362 |
| 14 | SeqId_3327_27 | | Netrin-4 | NET4 | -0.5540319 |
| 15 | SeqId_5731_1 | | Serine protease inhibitor Kazal-type 6 | ISk6 | 0.55237444 |
| 16 | SeqId_9889_42 | | Actin filament-associated protein 1-like 1 | AF1L1 | -0.5422456 |
| 17 | SeqId_5307_12 | | Coagulation factor IX | Coagulation Factor IXab | 0.52424523 |
| 18 | SeqId_7009_8 | | B-cell differentiation antigen CD72 | CD72 | -0.5158859 |
| 19 | SeqId_9266_1 | | Transmembrane and ubiquitin-like domain-containing protein 2 | sTREM-1 | 0.49286769 |
| 20 | SeqId_4496_60 | | Macrophage metalloelastase | MMP-12 | 0.48625879 |
| ^a^Proteins were ranked by the absolute weights. | | | | | |
