# Supplementary material for: Development, characterization, and replication of proteomic aging clocks: Analysis of 2 population-based cohorts
Source: PLoS Med. 2024 Sep 24;21(9):e1004464. doi: 10.1371/journal.pmed.1004464 (PMC11460707; doi:10.1371/journal.pmed.1004464)
Supplement: S15 Table — (DOCX) [file pmed.1004464.s022.docx]

**S15 Table. The association between age acceleration for the midlife ARIC PAC and published PACs and mortality stratified by sex, race, and chronological age (in tertiles); ARIC (1990-2019)**

| *Stratified by sex* | | | | | | | | |
| --- | --- | --- | --- | --- | --- | --- | --- | --- |
|  |  | No. of participants | No. of events | Total person-years | midlife ARIC PAC  HR (95% CI)^a^ | midlife Lehallier’s PAC  HR (95% CI)^a^ | midlife Tanaka’s PAC  HR (95% CI)^a^ | midlife Sathyan’s PAC  HR (95% CI)^a^ |
|  |  |  |  |  | per 1 SD^b^ | per 1 SD^b^ | per 1 SD^b^ | per 1 SD^b^ |
| All-cause mortality | Female | 4,789 | 2625 | 103,818 | 1.37 (1.31, 1.43), p<0.001 | 1.33 (1.28, 1.39), p<0.001 | 1.29 (1.24, 1.35), p<0.001 | 1.34 (1.28, 1.4), p<0.001 |
|  | Male | 3,979 | 2669 | 78,812 | 1.39 (1.33, 1.46), p<0.001 | 1.35 (1.29, 1.41), p<0.001 | 1.33 (1.27, 1.39), p<0.001 | 1.39 (1.34, 1.45), p<0.001 |
|  | P-interaction |  |  |  | 0.476 | 0.887 | 0.673 | 0.312 |
| CVD mortality (Fine and Gary model) | Female | 4,789 | 843 | 103,818 | 1.23 (1.13, 1.32), p<0.001 | 1.17 (1.09, 1.26), p<0.001 | 1.12 (1.04, 1.22), p<0.001 | 1.20 (1.11, 1.30), p<0.001 |
|  | Male | 3,979 | 891 | 78,812 | 1.19 (1.10, 1.28), p<0.001 | 1.20 (1.12, 1.29), p<0.001 | 1.15 (1.07, 1.24), p<<0.001 | 1.22 (1.14, 1.31), p<0.001 |
|  | P-interaction |  |  |  | 0.748 | 0.213 | 0.342 | 0.638 |
| cancer mortality (Fine and Gray model) | Female | 4,789 | 721 | 103,818 | 1.02 (0.94, 1.11),  P=0.519 | 1.08 (0.99, 1.17), p=0.208 | 1.05 (0.96, 1.14), p=0.203 | 0.98 (0.90, 1.06), p=0.553 |
|  | Male | 3,979 | 795 | 78,812 | 1.06 (0.97, 1.15),  P=0.382 | 1.03 (0.94, 1.12), p=0.103 | 1.05 (0.96, 1.15), p=0.517 | 1.04 (0.96, 1.13), p=0.312 |
|  | P-interaction |  |  |  | 0.863 | 0.112 | 0.448 | 0.581 |
| *Stratified by race* | | | | | | | | |
|  |  | No. of participants | No. of events | Total person-years | midlife ARIC PAC  HR (95% CI)^a^ | midlife Lehallier’s PAC  HR (95% CI)^a^ | midlife Tanaka’s PAC  HR (95% CI)^a^ | midlife Sathyan’s PAC  HR (95% CI)^a^ |
|  |  |  |  |  | per 1 SD^c^ | per 1 SD^c^ | per 1 SD^c^ | per 1 SD^c^ |
| All-cause mortality | White | 6,395 | 3859 | 136,883 | 1.36 (1.31, 1.41), p<0.001 | 1.34 (1.30, 1.39), p<0.001 | 1.31 (1.26, 1.35), p<0.001 | 1.36 (1.31, 1.41), p<0.001 |
|  | Black | 2,373 | 1435 | 45,747 | 1.40 (1.32, 1.50), p<0.001 | 1.32 (1.24, 1.40), p<0.001 | 1.31 (1.23, 1.39), p<0.001 | 1.35 (1.28, 1.43), p<0.001 |
|  | P-interaction |  |  |  | 0.248 | 0.941 | 0.187 | 0.823 |
| CVD mortality (Fine and Gray model) | White | 6,395 | 1168 | 136,883 | 1.23 (1.15, 1.30), p<0.001 | 1.19 (1.12, 1.26), p<0.001 | 1.16 (1.09, 1.23), p<0.001 | 1.22 (1.15, 1.30), p<0.001 |
|  | Black | 2,373 | 566 | 45,747 | 1.17 (1.06, 1.30), p<0.001 | 1.18 (1.07, 1.31), p<0.001 | 1.10 (0.99, 1.22), p<0.001 | 1.16 (1.05, 1.28), p<0.001 |
|  | P-interaction |  |  |  | 0.562 | 0.963 | 0.62 | 0.667 |
| cancer mortality (Fine and Gray model) | White | 6,395 | 1138 | 136,883 | 1.04 (0.98, 1.12), p=0.431 | 1.07 (1.00, 1.15), p=0.173 | 1.06 (0.99, 1.14), p=0.218 | 1.03 (0.97, 1.10), p=0.348 |
|  | Black | 2,373 | 378 | 45,747 | 1.05 (0.92, 1.19), p=0.346 | 1.01 (0.89, 1.14), p=0.282 | 1.01 (0.88, 1.15), p=0.742 | 0.97 (0.86, 1.09), p=0.566 |
|  | P-interaction |  |  |  | 0.912 | 0.376 | 0.819 | 0.445 |
| *Stratified by chronological age (in tertiles)* | | | | | | | | |
|  |  | No. of participants | No. of events | Total person-years | midlife ARIC PAC  HR (95% CI)^a^ | midlife Lehallier’s PAC  HR (95% CI)^a^ | midlife Tanaka’s PAC  HR (95% CI)^a^ | midlife Sathyan’s PAC  HR (95% CI)^a^ |
|  |  |  |  |  | per 1 SD^d^ | per 1 SD^d^ | per 1 SD^d^ | per 1 SD^d^ |
| All-cause mortality | 47 – 54 years | 2,971 | 1,135 | 70,088 | 1.47 (1.37, 1.57), p<0.001 | 1.39 (1.30, 1.49), p<0.001 | 1.35 (1.26, 1.45), p<0.001 | 1.33 (1.24, 1.43), p<0.001 |
|  | 55 – 60 years | 2,675 | 1,577 | 56,693 | 1.35 (1.27, 1.42), p<0.001 | 1.33 (1.26, 1.41), p<0.001 | 1.27 (1.20, 1.35), p<0.001 | 1.31 (1.24, 1.39), p<0.001 |
|  | 61 – 70years | 3,122 | 2,582 | 55,849 | 1.38 (1.32, 1.45), p<0.001 | 1.33 (1.27, 1.39), p<0.001 | 1.33 (1.27, 1.39), p<0.001 | 1.40 (1.34, 1.45), p<0.001 |
|  | P-interaction |  |  |  | <0.001 | 0.053 | 0.432 | 0.345 |
| CVD mortality (Fine and Gray model) | 47 – 54 years | 2,971 | 342 | 70,088 | 1.45 (1.28, 1.64), p<0.001 | 1.34 (1.20, 1.51), p<0.001 | 1.31 (1.16, 1.49), p<0.001 | 1.39 (1.22, 1.57), p<0.001 |
|  | 55 – 60 years | 2,675 | 574 | 56,693 | 1.11 (1.01, 1.23), p=0.002 | 1.11 (1.01, 1.22), p<0.001 | 1.10 (1.00, 1.21), p=0.110 | 1.15 (1.04, 1.27), p=0.005 |
|  | 61 – 70years | 3,122 | 878 | 55,849 | 1.19 (1.10, 1.28), p<0.001 | 1.18 (1.09, 1.27), p<0.001 | 1.11 (1.03, 1.20), p<0.001 | 1.20 (1.12, 1.30), p<0.001 |
|  | P-interaction |  |  |  | <0.001 | 0.004 | 0.001 | 0.031 |
| Cancer mortality (Fine and Gray model) | 47 – 54 years | 2,971 | 375 | 70,088 | 1.15 (1.02, 1.29), p=0.011 | 1.14 (1.01, 1.28), p=0.021 | 1.10 (0.97, 1.25), p=0.360 | 1.02 (0.89, 1.16), p=0.790 |
|  | 55 – 60 years | 2,675 | 495 | 56,693 | 0.97 (0.87, 1.08), p=0.943 | 1.03 (0.93, 1.14), p=0.165 | 0.98 (0.88, 1.10), p=0.983 | 1.05 (0.95, 1.16), p=0.382 |
|  | 61 – 70years | 3,122 | 646 | 55,849 | 1.02 (0.93, 1.13), p=0.800 | 1.00 (0.91, 1.10), p=0.634 | 1.06 (0.96, 1.17), p=0.210 | 1.00 (0.91, 1.08), p=0.895 |
|  | P-interaction |  |  |  | <0.001 | 0.154 | 0.589 | 0.523 |
| Abbreviations: PAC – proteomic aging clock; SD – standard deviation; BMI – body mass index; CVD – cardiovascular disease; eGFR – estimated glomerular filtration rate; HR – hazard ratio; CI – confidence interval. | | | | | | | | |
| ^a^The Model was adjusted for chronological age, sex, joint terms for race and study center (Black participants from Mississippi; Black participants from any other centers; White participants from Maryland; White participants from North Carolina; and White participants from Minnesota), education, BMI, smoking status, pack-years of smoking, alcohol intake, physical activity (at Visit 1), hormone replacement therapy (in females), diabetes, hypertension, CVD, and eGFR at Visit 2. | | | | | | | | |
| ^b^SDs for age acceleration across sex were: midlife ARIC PAC=3.02 and 2.85 years for females and males, respectively; midlife Lehallier’s PAC=3.04 and 2.95 years for females and males, respectively; midlife Tanaka’s PAC=3.17 and 3.13 years for females and males, respectively; and midlife Sathyan’s PAC=2.67 and 2.60 years for females and males, respectively. | | | | | | | | |
| ^c^SDs for age acceleration across race were: midlife ARIC PAC=2.75 and 3.40 years for White and Black participants, respectively; midlife Lehallier’s PAC=2.84 and 3.40 years for White and Black participants, respectively; midlife Tanaka’s PAC=2.95 and 3.61 years for White and Black participants, respectively; and midlife Sathyan’s PAC=2.50 and 2.99 years for White and Black participants, respectively. | | | | | | | | |
| ^d^SDs for age acceleration across chronological age groups were: midlife ARIC PAC=2.79, 2.94 and 3.09 years for participants aged 47-54 years, 55-60 years, and 61-70 years, respectively; midlife Lehallier’s PAC=2.89, 3.01 and 3.09 years for participants aged 47-54 years, 55-60 years, and 61-70 years, respectively; midlife Tanaka’s PAC=3.01, 3.08, and 3.34 years for participants aged 47-54 years, 55-60 years, and 61-70 years, respectively; and midlife Sathyan’s PAC = 2.52, 2.61, and 2.76 years for participants aged 47-54 years, 55-60 years, and 61-70 years, respectively. | | | | | | | | |
