# Supplementary material for: Development, characterization, and replication of proteomic aging clocks: Analysis of 2 population-based cohorts
Source: PLoS Med. 2024 Sep 24;21(9):e1004464. doi: 10.1371/journal.pmed.1004464 (PMC11460707; doi:10.1371/journal.pmed.1004464)
Supplement: S16 Table — (DOCX) [file pmed.1004464.s023.docx]

**S16 Table. The associations of age acceleration for the late-life ARIC PAC and published PACs with mortality stratified by sex, race, and chronological age (in tertiles); ARIC (2011-2019)**

| *Stratified by sex* | | | | | | | |
| --- | --- | --- | --- | --- | --- | --- | --- |
|  | No. of participants | No. of event | Total person-years | late-life ARIC PAC  HR (95% CI)^a^  per 1 SD^b^ | late-life Lehallier’s PAC  HR (95% CI)^a^  per 1 SD^b^ | late-life Tanaka’s PAC  HR (95% CI)^a^  per 1 SD^b^ | late-life Sathyan’s PAC  HR (95% CI)^a^  per 1 SD^b^ |
| All-cause mortality | | | | | | | |
| Female | 2,565 | 536 | 16,704 | 1.70 (1.51, 1.92), p<0.001 | 1.65 (1.46, 1.87), p<0.001 | 1,64 (1.45, 1.86), p<0.001 | 1.72 (1.52, 1.94), p<0.001 |
| Male | 1,988 | 587 | 12,652 | 1.62 (1.44, 1.82), p<0.001 | 1.54 (1.37, 1.73), p<0.001 | 1.43 (1.27, 1.61), p<0.001 | 1.57 (1.41, 1.76), p<0.001 |
| P-interaction |  |  |  | 0.109 | 0.104 | 0.009 | 0.043 |
| CVD mortality (Fine and Gray model) | | | | | | | |
| Female | 2,565 | 163 | 16,704 | 1.30 (1.03, 1.63), p=0.025 | 1.36 (1.07, 1.74), p=0.014 | 1.25 (0.97, 1.60), p=0.079 | 1.32 (1.03, 1.69), p=0.023 |
| Male | 1,988 | 185 | 12,652 | 1.42 (1.18, 1.72), p<0.001 | 1.40 (1.14, 1.71), p=0.001 | 1.39 (1.16, 1.66), p<0.001 | 1.48 (1.25, 1.74), p<0.001 |
| P-interaction |  |  |  | 0.342 | 0.245 | 0.378 | 0.503 |
| Cancer mortality (Fine and Gray model) | | | | | | | |
| Female | 2,565 | 129 | 16,704 | 1.68 (1.25, 2.04), p<0.001 | 1.50 (1.18, 1.90), p<0.001 | 1.58 (1.23, 2.03), p<0.001 | 1.70 (1.37, 2.10), p<0.001 |
| Male | 1,988 | 149 | 12,652 | 0.95 (0.75, 1.20), p=0.685 | 0.99 (0.80, 1.22), p=0.921 | 0.93 (0.74, 1.17), p=0.535 | 0.95 (0.76, 1.17), p=0.672 |
| P-interaction |  |  |  | 0.021 | 0.038 | 0.033 | 0.004 |
| *Stratified by race* | | | | | | | |
|  | No. of participants | No. of event | Total person-years | late-life ARIC PAC  HR (95% CI)^a^  per 1 SD^c^ | late-life Lehallier’s PAC  HR (95% CI)^a^  per 1 SD^c^ | late-life Tanaka’s PAC  HR (95% CI)^a^  per 1 SD^c^ | late-life Sathyan’s PAC  HR (95% CI)^a^  per 1 SD^c^ |
| All-cause mortality |  |  |  |  |  |  |  |
| White | 3,655 | 935 | 24,637 | 1.67 (1.53, 1.82), p<0.001 | 1.56 (1.43, 1.71), p<0.001 | 1.51 (1.38, 1.65), p<0.001 | 1.67 (1.54, 1.82), p<0.001 |
| Black | 898 | 188 | 4,718 | 1.57 (1.23, 2.00), p<0.001 | 1.71 (1.34, 2.18), p<0.001 | 1.63 (1.26, 2.09), p<0.001 | 1.36 (1.07, 1.72), p<0.001 |
| P-interaction |  |  |  | 0.623 | 0.201 | 0.156 | 0.602 |
| CVD mortality (Fine and Gray model) | | | | | | | |
| White | 3,655 | 274 | 24,637 | 1.29 (1.10, 1.52), p<0.001 | 1.28 (1.09, 1.50), p=0.002 | 1.25 (1.07, 1.45), p=0.002 | 1.37 (1.19, 1.57), p<0.001 |
| Black | 898 | 74 | 4,718 | 2.03 (1.36, 3.06), p=0.001 | 2.06 (1.27, 3.36), p=0.002 | 2.03 (1.30, 3.17), p<0.001 | 1.70 (1.11, 2.60), p=0.014 |
| P-interaction |  |  |  | 0.774 | 0.332 | 0.389 | 0.953 |
| Cancer mortality (Fine and Gray model) | | | | | | | |
| White | 3,655 | 233 | 24,637 | 1.24 (1.03, 1.48), p=0.015 | 1.22 (1.03, 1.44), p=0.006 | 1.22 (1.01, 1.48), p=0.016 | 1.30 (1.10, 1.53), p=0.042 |
| Black | 898 | 45 | 4,718 | 0.82 (0.50, 1.36), p=0.311 | 0.85 (0.56, 1.28), p=0.270 | 0.77 (0.46, 1.27), p=0.295 | 0.76 (0.47, 1.22), p=0.110 |
| P-interaction |  |  |  | 0.643 | 0.872 | 0.738 | 0.826 |
| Stratified by chronological age (in tertiles) | | | | | | | |
|  | No. of participants | No. of event | Total person-years | late-life ARIC PAC  HR (95% CI)^a^  per 1 SD^d^ | late-life Lehallier’s PAC  HR (95% CI)^a^  per 1 SD^d^ | late-life Tanaka’s PAC  HR (95% CI)^a^  per 1 SD^d^ | late-life Sathyan’s PAC  HR (95% CI)^a^  per 1 SD^d^ |
| All-cause mortality |  |  |  |  |  |  |  |
| 67-72 | 1,432 | 177 | 9757 | 1.61 (1.31, 1.99), p<0.001 | 1.63 (1.27, 2.10), p<0.001 | 1.47 (1.14, 1.89), p<0.001 | 1.59 (1.24, 2.04), p<0.001 |
| 73-78 | 1,678 | 347 | 10,975 | 1.71 (1.45, 2.01), p<0.001 | 1.64 (1.41, 1.91), p<0.001 | 1.64 (1.40, 1.92), p<0.001 | 1.63 (1.41, 1.89), p<0.001 |
| 79-90 | 1,443 | 599 | 8,625 | 1.68 (1.50, 1.90), p<0.001 | 1.52 (1.35, 1.70), p<0.001 | 1.43 (1.27, 1.60), p<0.001 | 1.62 (1.45, 1.80), p<0.001 |
| P-interaction |  |  |  | 0.621 | 0.478 | 0.189 | 0.643 |
| CVD mortality (Fine and Gray model) | | | | | | | |
| 67-72 | 1,432 | 57 | 9757 | 1.89 (1.22, 2.93), p=0.021 | 1.35 (0.76, 2.39), p=0.315 | 1.19 (0.69, 2.07), p=0.531 | 1.24 (0.71, 2.16), p=0.455 |
| 73-78 | 1,678 | 98 | 10,975 | 1.40 (1.01, 1.94), p=0.004 | 1.57 (1.18, 2.09), p=0.002 | 1.50 (1.08, 2.08), p=0.017 | 1.54 (1.18, 2.01), p=0.002 |
| 79-90 | 1,443 | 193 | 8,625 | 1.32 (1.08, 1.62), p=0.042 | 1.25 (1.02, 1.53), p=0.032 | 1.21 (1.00, 1.46), p=0.052 | 1.36 (1.13, 1.63), p=0.001 |
| P-interaction |  |  |  | 0.036 | 0.212 | 0.253 | 0.999 |
| Cancer mortality (Fine and Gray model) | | | | | | | |
| 67-72 | 1,432 | 57 | 9757 | 1.25 (0.80, 1.95), p=0.510 | 1.63 (1.05, 2.53), p=0.028 | 1.49 (0.86, 2.58), p=0.165 | 1.45 (0.78, 2.67), p=0.241 |
| 73-78 | 1,678 | 101 | 10,975 | 1.18 (0.87, 1.59), p=0.115 | 1.25 (0.94, 1.66), p=0.125 | 1.32 (0.99, 1.77), p=0.063 | 1.27 (0.98, 1.65), p=0.073 |
| 79-90 | 1,443 | 120 | 8,625 | 1.19 (0.92, 1.54), p=0.193 | 1.11 (0.88, 1.40), p=0.397 | 1.06 (0.80, 1.40), p=0.702 | 1.21 (0.95, 1.54), p=0.135 |
| P-interaction |  |  |  | 0.703 | 0.445 | 0.556 | 0.921 |
| Abbreviations: BMI – body mass index; CVD – cardiovascular disease; eGFR - estimated glomerular filtration rate; HR – hazard ratio; CI – confidence interval. | | | | | | | |
| ^a^The Model was adjusted for chronological age, sex, joint terms for race and study center (Black participants from Mississippi; Black participants from any other centers; White participants from Maryland; White participants from North Carolina; and White participants from Minnesota), education, BMI, smoking status, pack-years of smoking, alcohol intake, physical activity, diabetes, hypertension, CVD, and eGFR at Visit 5. | | | | | | | |
| ^b^SDs for age acceleration across sex were: late-life ARIC PAC=2.56 and 2.68 years for females and males, respectively; late-life Lehallier’s PAC=2.50 and 2.57 years for females and males, respectively; late-life Tanaka’s PAC=2.81 and 2.93 years for females and males, respectively; late-life Sathyan’s PAC=3.16 and 3.31 years for females and males, respectively. | | | | | | | |
| ^c^SDs for age acceleration across race were: late-life ARIC PAC=2.50 and 3.03 years for White and Black participants, respectively; late-life Lehallier’s PAC=2.49 and 2.77 years for White and Black participants, respectively; late-life Tanaka’s PAC=2.81 and 3.21 years for White and Black participatns, respectively; late-life Sathyan’s PAC=3.15 and 3.51 years for White and Black participants, respectively. | | | | | | | |
| ^d^ SDs for age acceleration across chronological age groups were: late-life ARIC PAC= 2.42, 2.66, and 2.74 years for participants aged 67-72, 73-78, and 79-90 years, respectively; late-life Lehallier’s PAC=2.46, 2.48, and 2.66 years for participants aged 67-72, 73-78, and 79-90 years, respectively; late-life Tanaka’s PAC= 2.76, 2.81, and 3.02 years participants aged 67-72, 73-78, and 79-90 years, respectively; late-life Sathyan’s PAC=3.07, 3.18, and 3.40 years for participants aged 67-72, 73-78, and 79-90 years, respectively. | | | | | | | |
